# A systematic review and network meta-analysis of single nucleotide polymorphisms associated with pancreatic cancer risk

**DOI:** 10.18632/aging.104128

**Published:** 2020-11-20

**Authors:** Zhuo-Miao Ye, Li-Juan Li, Ming-Bo Luo, Hong-Yuan Qing, Jing-Hui Zheng, Chi Zhang, Yun-Xin Lu, You-Ming Tang

**Affiliations:** 1 Department of Oncology, Xiangya Hospital, Central South University, Changsha 410008, China; Ruikang School of Clinical Medicine, Guangxi University of Chinese Medicine, Nanning 530001, China; 2 Ruikang School of Clinical Medicine, Guangxi University of Chinese Medicine, Nanning 530001, China; 3 The First Clinical Faculty of Guangxi University of Chinese Medicine, Guangxi University of Chinese Medicine, Nanning 530222, China; 4 Ruikang School of Clinical Medicine, Guangxi University of Chinese Medicine, Nanning 530001, China; 5 Department of Cardiology, Ruikang Hospital Affiliated to Guangxi University of Chinese Medicine, Nanning 530011, China; 6 Graduate School, Guangxi University of Chinese Medicine, Nanning 530001, Guangxi, China; 7 Department of Oncology, Ruikang Hospital Affiliated to Guangxi University of Chinese Medicine, Nanning 530011, China; 8 Department of Gastroenterology, Ruikang Hospital Affiliated to Guangxi University of Chinese Medicine, Nanning, China

**Keywords:** pancreatic cancer, single nucleotide polymorphisms, network meta-analysis, FPRP

## Abstract

In this meta-analysis, we systematically investigated the correlation between single nucleotide polymorphisms (SNPs) and pancreatic cancer (PC) risk. We searched PubMed, Network Science, EMBASE, Cochrane Library, China National Knowledge Infrastructure (CNKI), China Science and Technology Periodical Database (VIP), and Wanfang databases up to January 2020 for studies on PC risk-associated SNPs. We identified 45 case-control studies (36,360 PC patients and 54,752 non-cancer individuals) relating to investigations of 27 genes and 54 SNPs for this meta-analysis. Direct meta-analysis followed by network meta-analysis and Thakkinstian algorithm analysis showed that homozygous genetic models for *CTLA-4* rs231775 (OR =0.326; 95% CI: 0.218-0.488) and *VDR* rs2228570 (OR = 1.976; 95% CI: 1.496-2.611) and additive gene model for *TP53* rs9895829 (OR = 1.231; 95% CI: 1.143-1.326) were significantly associated with PC risk. *TP53* rs9895829 was the most optimal SNP for diagnosing PC susceptibility with a false positive report probability < 0.2 at a stringent prior probability value of 0.00001. This systematic review and meta-analysis suggest that *TP53* rs9895829, *VDR* rs2228570, and *CTLA-4* rs231775 are significantly associated with PC risk. We also demonstrate that *TP53* rs9895829 is a potential diagnostic biomarker for estimating PC risk.

## INTRODUCTION

Pancreatic cancer (PC) is the eighth leading cause of cancer-related deaths worldwide with a 1-year survival rate of less than 5% [1]. The risk factors associated with pancreatic cancer include smoking, heavy alcohol drinking, diabetes, obesity, chronic pancreatitis, family history, and genetic factors [2]. Genetic mutations in *TERT, UGT2B4, XRCC4, XPC, SLC22A3, NR5A2, ABO* and *XPD* genes are associated with susceptibility to pancreatic cancer [3–10]. Single nucleotide polymorphisms (SNPs) in several genes correlate with increased risk of pancreatic cancer [11]. SNPs in protein-coding genes and non-coding RNAs are the most common type of gene mutations implicated in several human disease. Specific SNPs are associated with increased or decreased risk of multiple cancer types because of a genetic phenomenon called linkage disequilibrium (LD) [12]. Variants in insulin-like growth factor, platelet-derived growth factor subunit B, atopy-related immunologic candidate genes, taste-related genes, and inflammatory genes are associated with pancreatic cancer (PC) risk [5, 11, 13–16]. However, the results of many SNP-related studies are often inconclusive because of small sample sizes. Therefore, systematic review of multiple studies is required to analyze the relationship between pancreatic cancer and SNPs [17–20]. There are very few systematic reviews regarding the relationship between SNPs and pancreatic cancer. Therefore, we performed this meta-analysis to identify prominent SNPs associated with greater PC risk. We then selected the most suitable genetic model by comparing data for these PC-related SNPs from network meta-analysis and Thakkinstian algorithm. We then evaluated the reliability of the meta-analysis results using the false positive report probability (FPRP) to determine the most strongly associated SNPs with pancreatic cancer susceptibility.

## RESULTS

### Description of included studies

This study included 45 studies with 36,360 PC patients and 54,752 non-cancer controls. Supplementary Table 2 shows the data characteristics of the meta-analysis. Initial screening identified 178 genes and 419 SNPs in the included studies, but, only 27 genes and 54 SNPs met the final selection criteria. The genes and SNPs identified in the 45 studies are shown in the Supplementary Table 2. See Table 1 for more details. A total of 45 articles were included [2, 3, 5, 9, 21–60]. The results of the quality evaluation of the included studies are shown in Supplementary Table 1. The evaluation criteria of this study include the following nine aspects: (1) whether to describe genotyping methods; (2) Whether to describe the population stratification method; (3) Whether to describe genotype inference method; (4) Whether the genotype distribution of the control group conforms to HWE; (5) Whether to emphasize the repeatability of research; (6) Whether to describe the inclusion and exclusion criteria and matching methods for the research objects; (7) Whether the statistical method and software version are explained; (8) Correlation judgment method; (9) Whether the data is sufficient

**Table 1:** Included SNPS and their corresponding literatures.

**Gene**	**SNP**
X-ray repair cross-complementing protein 4 (XRCC4)	rs2075685, rs1805377^5, 24^
X-ray repair cross-complementing protein 1 (XRCC1)	rs25487, rs1799782^25, 26^
xeroderma pigmentosum group C (XPC)	rs3731055, rs2228001, rs2228000, rs2607775^27-29^
vascular endothelial growth factor (VEGF)	rs833061, rs2010963^30, 31^
vitamin D receptor (VDR)	rs2228570, rs1544410^32, 33^
Tumor protein p53 (TP53)	rs9895829^34, 35^
fat mass and obesity-associated (FTO)	rs9939609^36, 37^
excision repair cross-complementary group 1 (ERCC1)	rs11615, rs3212986 ^37,38^
excision repair cross complementation group 2 (ERCC2)	rs13181^29, 38^
excision repair cross complementation group 4 (ERCC4)	rs6498486^29, 38^
cytotoxic T-lymphocyte antigen-4 (CTLA-4)	rs231775^39, 40^
cyclooxygenase-2 (COX-2)	rs20417^41, 42^
cyclin dependent kinase inhibitor 2A/B (CDKN2A/B)	rs3731257, rs3731249, rs3731239,rs3731211,rs3218009, rs3217992, rs3217986, rs2811710, rs2811708,rs2518719, rs11515, rs1063192^43^
ABO blood groups (ABO)	rs657152, rs505922, rs495828^44-47^
Telomerase reverse transcriptase (TERT)	rs2736098, rs401681, rs2853677^45, 48-50^
sterile alpha motif domain containing 12-TNF receptor superfamily member 11 (SAMD12–TNFRSF11B)	rs11988997^51^
Serine protease 1/2 (PRSS1-PRSS2)	rs10273639^51, 52^
KIAA1462-mitochondrial poly(A) polymerase (KIAA1462-MTPAP)	rs2995271^51^
MUM1-like 1(MUM1L1-CXorf57)	rs379742^51^
MORC family CW-type zinc finger 4(MORC4)	rs12837024^51^
8-oxoguanine DNA glycosylase 1 (OGG1)	rs1052133^25, 53^
strand of HoxC gene (HOTAIR)	rs4759314^54, 55^
epithelial cadherin (E-cadherin)	rs16260^56, 57^
Tumor necrosis factor-α (TNF-α)	rs1800629^58-61^
methylenetetrahydrofolate reductase (MTHFR)	rs1801133^62, 63^
Insulin-like growth factor 1 (IGF-1)	rs2288378, rs5742714^64, 65^
hypoxia inducible factor-1alpha (HIF-1α)	rs11549467, rs11549465^66, 67^

### Pairwise meta-analysis

Supplementary Table 3 shows the results of the direct meta-analysis to determine the correlation between 54 SNPs and PC risk. We evaluated 6 genetic models for all SNPs to determine the most optimal genetic model that shows correlation with PC risk. The GG and AA genotypes of *TP53* rs9895829 showed significant correlation with higher PC risk compared to the GA genotype (GG+AA vs. GA: pooled OR =1.231, 95% CI: 1.143-1.326). The CC and GG genotypes of *COX2*-765 showed significant correlation with reduced PC risk compared to the CG allele (CC+GG vs. CG: pooled OR = 0.398, 95% CI: 0.273-0.579). According to the fixed effects model, AA and GG genotypes of *HIF-1α* rs11549467 correlated with increased PC risk compared to the AG genotype (AA+GG vs. AG: pooled OR = 0.343, 95% CI: 0.216-0.545). According to the fixed effects model, the CC genotype of *VDR* rs2228570 was associated with increased PC risk compared to the TT genotype (CC vs. TT: pooled OR =0.326 95% CI: 0.218-0.488). The homozygous AA genotype of *CTLA-4* rs231775 was associated with increased PC risk compared to the GG genotype (AA vs. GG: pooled OR = 1.976, 95% CI: 1.496-2.611). The fixed effects model showed that the TT and TC genotypes of *MTHFR* rs1801133 showed increased PC risk than the CC genotype (TT+TC vs. CC; pooled OR = 1.905, 95% CI: 1.355-2.677). In addition to this, direct meta-analysis of other meaningful models showed the following in Table 2.

**Table 2:** Results of direct meta-analysis of SNPS in different gene models.

**Gene**	**Genetic model**	**OR 95%CI**
*TP53* rs9895829		
*G vs A*	Allele model	0.815(0.759-0.874)
GA vs AA	Heterozygous gene model	0.811(0.754-0.874)
GG+GA vs AA	Dominant gene model	0.808(0.751-0.870)
GG+AA vs GA	Additive gene model	1.231(1.143-1.326)
*COX-2* -765		
C vs G	Allele model	2.439(1.687-3.524)
CG vs GG	Heterozygous gene model	2.514(1.728-3.657)
CC+CG vs GG	Dominant gene model	2.514(1.728-3.657)
CC+GG vs CG	Additive gene model	0.398(0.273-0.579)
*HIF-1α* rs11549467		
A vs G	Allele model	3.075(1.981-4.775)
AG vs GG	Heterozygous gene model	2.946(1.853-4.683)
AA+AG vs GG	Dominant gene model	3.142(1.987-4.970)
AA+GG vs AG	Additive gene model	0.343(0.216-0.545)
*VDR* rs2228570		
C vs T	Allele model	0.530(0.433-0.649)
CC vs TT	Homozygous gene model	0.326(0.218-0.488)
CC vs CT+TT	Recessive gene model	0.403(0.297-0.547)
CC+CT vs TT	Dominant gene model	0.571(0.363-0.737)
CC+TT vs TC	Additive gene model	0.667(0.503-0.884)
*CTLA-4* rs231775		
A vs G	Allele model	1.378(1.221-1.555)
AA vs GG	Homozygous gene model	1.976(1.496-2.611)
AG vs GG	Heterozygous gene model	1.391(1.167-1.658)
AA vs AG+GG	Recessive gene model	1.668(1.286-2.164)
AA+AG vs GG	Dominant gene model	1.491(1.262-1.763)
AA+GG vs AG	Additive gene model	0.831(0.706-0.979)
*MTHFR* rs1801133		
T vs C	Allele model	1.674(1.337-2.095)
TT vs CC	Homozygous gene model	2.979(1.844-4.812)
TC vs CC	Heterozygous gene model	1.669(1.163-2.395)
TT vs TC+CC	Recessive gene model	2.096(1.397-3.146)
TT+TC vs CC	Dominant gene model	1.905(1.355-2.677)

### Network meta-analysis and Thakkinstian algorithm analysis of the most appropriate genetic models for SNPs associated with PC risk

**Figure 1:**
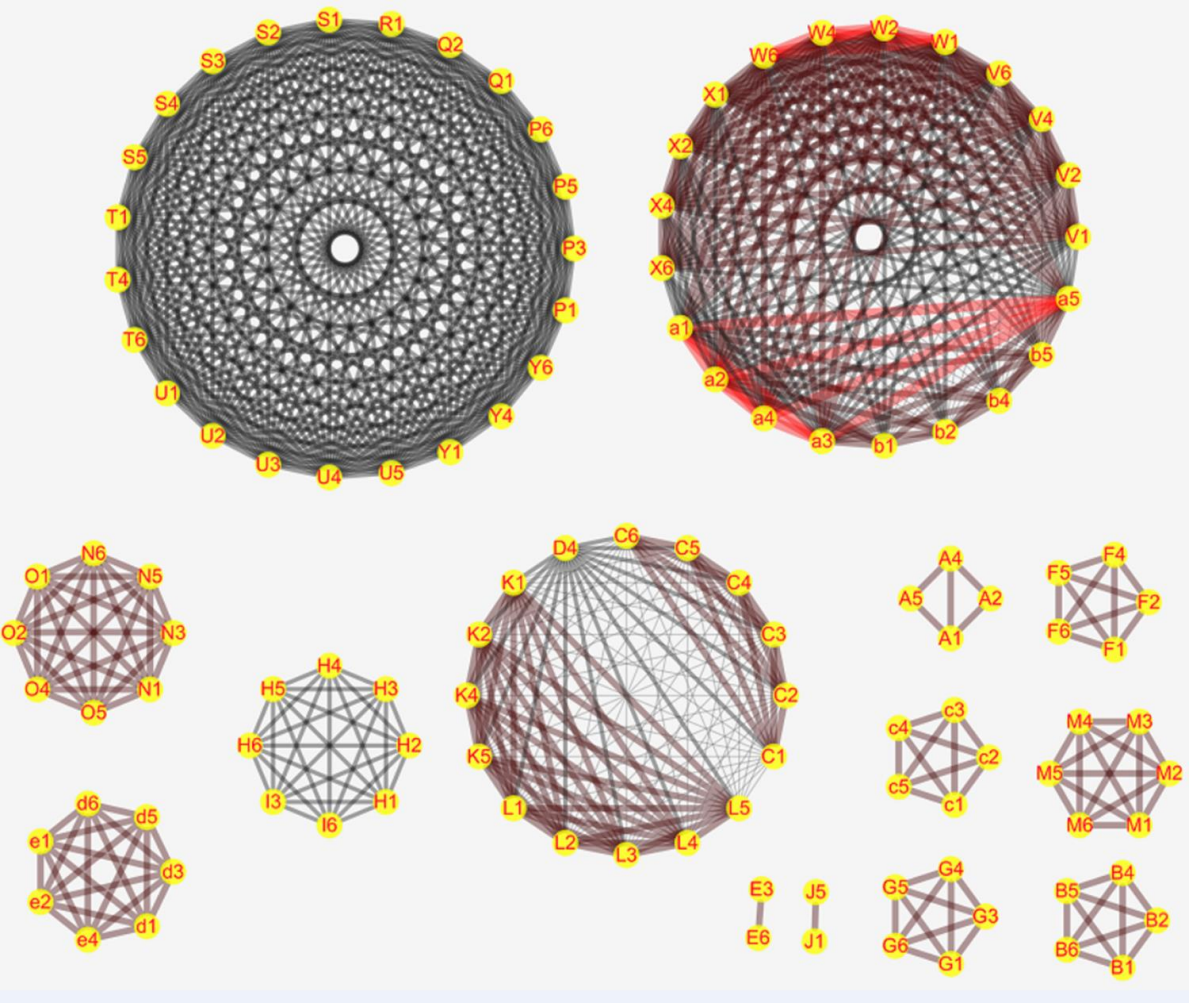
**Network meta-analysis results for the genetic models of the PC risk-related SNPs.** The figure shows the network meta-analysis results for the (1) Allele (2) Homozygous (3) Heterozygous (4) Dominant (5) Recessive and (6) Additive genetic models of the following SNPs: (C) *XPC* rs2607775; (D) *XPC* rs2228001; (K) *ERCC2* rs13181; (L) *ERCC1* rs3212986; (V) *ABO* rs657152; (W) *ABO* rs505922; (X) *ABO* rs495828; (V) *ABO* rs657152; (N) *COX2*-765; (O) *COX2*-1195; (H) *MUM1L1-CXorf57* rs379742; (I) *MORC4* rs12837024; (d) *HIF-1α* G1790A rs11549467; (e) *HIF-1α* C1772T rs11549465; (P) *CDKN2A/B* rs3731249; (Q) *CDKN2A/B* rs3731211; (R) *CDKN2A/B* rs3218009; (S) *CDKN2A/B* rs3217992; (T) *CDKN2A/B* rs2518719; (U) *CDKN2A/B* rs1063192; (Y) *CDKN2A/B* rs1063192; (A) *XRCC4* rs2075685; (B) *XRCC1* rs25487; (F) *VDR* rs2228570; (G) *TP53* rs9895829; (M) *CTLA-4* rs231775; (E) *VEGF* +405 rs2010963; (c) *MTHFR* rs1801133; (J) *FTO* rs9939609; (b) TERT rs2853677. D.

We performed network meta-analysis with the consistency model to compare the genetic models of different SNPs that show significant correlation with PC risk in order to select the most suitable genetic model for studying PC susceptibility. The results showed that some SNPs were linked to a network, whereas the others were linked only with their genetic models (Figure 1). There are altogether 14 networks, among which 6 networks are formed according to different SNPS, while the other 8 networks are formed within SNPS only with different models as nodes due to insufficient data. After comparing the genetic models with network meta-analysis and paired meta-analysis (Supplementary Tables 1–14, Supplementary Materials 1), we selected 14 SNPs (*COX2*-765, *HIF-1α* rs11549467, *VDR* rs2228570, *TP53* rs9895829, *CTLA-4* rs231775, *MTHFR* rs1801133, *ABO* rs495828, *FTO* rs9939609, *CDKN2A/B* rs2518719, *XRCC4* rs2075685, *XRCC1* rs25487, *XPC* rs2607775, *MORC4* rs12837024, *VEGF* +405G/C rs2010963, *MTHFR* rs1801133) and their the most suitable gene model. Based on the rank probabilities, the optimal models for most genes were either additive or dominant (Figure 1). The results of Thakkinstian analysis showed that the co-dominant model was most optimal for these 14 SNPs (Table 2). The prior probability FPRP values for these 14 SNPs are summarized in Table 2.

Both network meta-analysis and the Thakkinstian's criteria showed different optimal gene models for the 14 SNPs, but the model that showed significant correlation when the FPRP value was less than 0.2 was selected as the most optimal one to determine PC risk (Supplementary Table 3, Tables 2, 3). A SNP consists of a dominant allele (G) and a recessive allele (g). Pairwise differences of GG versus gg (D1), Gg versus gg (D2), and GG versus Gg (D3) were calculated as pooled OR1, OR2, and OR3, respectively, along with 95% CIs, in the pairwise meta-analysis. The most appropriate genetic model was determined to be: recessive model if OR1 = OR3 ≠ 1 and OR2 =1, dominant model if OR1 = OR2 ≠ 1 and OR3 =1, a complete over-dominant model if OR2 = 1/OR3 ≠1 and OR1 = 1, or codominant model if OR1 > OR2 > 1 and OR1 > OR3 > 1 (or if OR1 < OR2 < 1, and OR1 < OR3 < 1). For example, network meta-analysis and Thakkinstian's analysis of the HIF-1α rs11549467 SNP suggested that the additive and co-dominant gene models were most optimal, respectively. However, the FPRP value for the dominant gene model was below 0.2 and for the co-dominant model was above 0.2. Therefore, the additive gene model was considered as the best gene model for *HIF-1α* rs11549467 to determine PC risk. Similarly, we determined the most optimal genetic models to correlate with PC risk for *ABO* rs495828, *FTO* rs9939609, *CDKN2A/B* rs2518719, *XRCC4* rs2075685, *XRCC1* rs25487, *XPC* rs2607775, *MORC4* rs12837024, *VEGF* +405 rs2010963, and *MTHFR* rs1801133 using the criteria of FPRP value < 0.2 as indicating significant PC risk.

**Table 3:** Eleven snps’s best suitable gene model.

**Gene**		**OR 95%CI**	**Thakkinstian’s algorithm results**	**the value of FPRP#**	**Optimal genetic model**
ABO rs495828					
GGvsTT*		0.721(0.523-0.994)		0.869	
GGvsGT	D3	0.738(0.624-0.872)		0.039	
GTvsTT	D2	0.979(0.705-1.359)	codominant model	0.989	
GGvsTT	D1	0.721(0.523-0.994)		0.869	
COX-2 -765					
CC+GGvsCG*		2.514(1.728-3.657)		0.039	
CCvsCG	D3				
CGvsCC	D2		codominant model		Additive gene model
CCvsGG	D1				
FTO rs9939609					
AA+ATvsTT*		1.168(1.006-1.357)		0.808	
AAvsAT	D3	1.097(0.868-1.387)		0.978	
ATvsTT	D2	1.151(0.983-1.347)	codominant model	0.067	
AAvsTT	D1	1.194(0.939-1.518)		0.938	
HIF-1α rs11549467					
AA+GGvsAG*		0.343(0.216-0.545)		0.193	
AAvsAG	D3	29.4(1.119-772.373)		0.991	
AGvsGG	D2	2.946(1.853-4.683)	codominant model	0.184	Additive gene model
AAvsGG	D1	18.303(0.930-360.191)		0.991	
CDKN2A/B rs2518719					
GG+AAvsGA*		1.124(1.002-1.262)		0.826	
GGvsGA	D3	1.129(1.005-1.267)		0.795	
GAvsAA	D2	1.048(0.729-1.508)	codominant model	0.988	
GGvsAA	D1	1.183(0.831-1.685)		0.975	
XRCC4 rs2075685					
GG+GTvsTT*		0.695(0.534-0.904)		0.515	
GGvsGT	D3	0.739(0.565-0.967)		0.779	
GTvsTT	D2	0.781(0.589-1.036)	codominant model	0.908	
GGvsTT	D1	0.578(0.423-0.790)		0.238	
XRCC1 rs25487					
GG+GAvsAA*		0.627(0.401-0.980)		0.911	
GGvsGA	D3	0.718(0.558-0.923)		0.573	
GAvsAA	D2	0.767(0.479-1.230)	codominant model	0.974	
GGvsAA	D1	0.545(0.345-0.863)		0.83	
VDR rs2228570					
CC+CTvsTT*		0.571(0.363-0.737)		0.014	
CCvsCT	D3	0.442(0.319-0.612)		0.013	
CTvsTT	D2	0.737(0.504-1.076)	codominant model	0.942	Recessive gene model
CCvsTT	D1	0.326(0.218-0.488)		0.02	
TP53 rs9895829					
GG+AAvsGA*		1.231(1.143-1.326)		0.0001	
GGvsGA	D3	0.852(0.539-1.344)		0.983	
GAvsAA	D2	0.811(0.754-0.874)	codominant model	0.0001	Additive gene model
GGvsAA	D1	0.691(0.440-1.086)		0.951	
CTLA-4 rs231775					
AA+AGvsGG*		1.491(1.261-1.763)		0.001	
AAvsAG	D3	1.433(1.093-1.879)		0.593	
AGvsGG	D2	1.391(1.167-1.658)	codominant model	0.028	Dominant gene model
AAvsGG	D1	1.976(1.496-2.611)		0.006	
MORC4 rs12837024					
TT+CCvsTC*		1.176(1.029-1.344)		0.632	
TTvsTC	D3	1.225(1.035-1.449)		0.641	
TCvsCC	D2	0.860(0.751-0.986)	codominant model	0.752	
TTvsCC	D1	1.064(0.934-1.212)		0.972	
VEGF +405 rs2010963					
GG+CCvsGC*		2.838(1.765-4.563)		0.28	
GGvsGC	D3	2.033(1.185-3.488)		0.88	
GCvsCC	D2	0.265(0.075-0.937)	codominant model	0.981	
GGvsCC	D1	0.495(0.073-3.366)		0.992	
MTHFR rs1801133					
TT+TCvsCC*		1.905(1.355-2.677)		0.194	
TTvsTC	D3	1.218(0.409-3.621)		0.991	
TCvsCC	D2	1.669(1.163-2.395)	codominant model	0.657	Dominant gene model
TTvsCC	D1	2.979(1.844-4.812)		0.242	
XPC rs2607775					
GG+CCvsGC*		0.573(0.403-0.814)		0.483	
GGvsCG	D3	1.952(0.628-6.071)		0.987	
CGvsCC	D2	1.797(1.263-2.555)	codominant model	0.409	
GGvsCC	D1	3.523(1.176-10.551)		0.974	

Note:

1. *: This gene model is the most suitable gene model obtained by the network meta-analysis.

2. #: FPRP was used to detect the gene models obtained through the network meta-analysis and Thakkinstian’s algorithm respectively. The codominant gene model was replaced by the results of homozygous gene model and heterozygous gene model.

3. Optimal genetic model: the final genetic model was obtained after FPRP detection of two possible genetic models.

4. D1, D2, D3 were defined by Thakkinstian’s algorithm. D1: Heterozygous gene model; D2: Heterozygous gene model; D3: mutant homozygote vs heterozygote.

The additive gene models for *COX2-765, HIF-1α* rs11549467, and *TP53* rs9895829, the recessive gene model for *VDR* rs2228570 and TP53 rs9895829, the recessive gene model for *VDR* rs2228570, as well as dominant gene models for VDR rs2228570, *CTLA-4* rs231775 and *MTHFR* rs1801133 were the most optimal for determining PC susceptibility. Moreover, when the prior probability was 0.00001, the additive gene model for *TP53* rs9895829 showed a FPRP value below 0.2, whereas, the remaining showed FPRP values above 0.2. Therefore, we concluded that TP53 rs9895829 was the most optimal gene for predicting PC risk among all candidate genes and SNPs.

### Diagnostic meta-analysis

**Figure 2:**
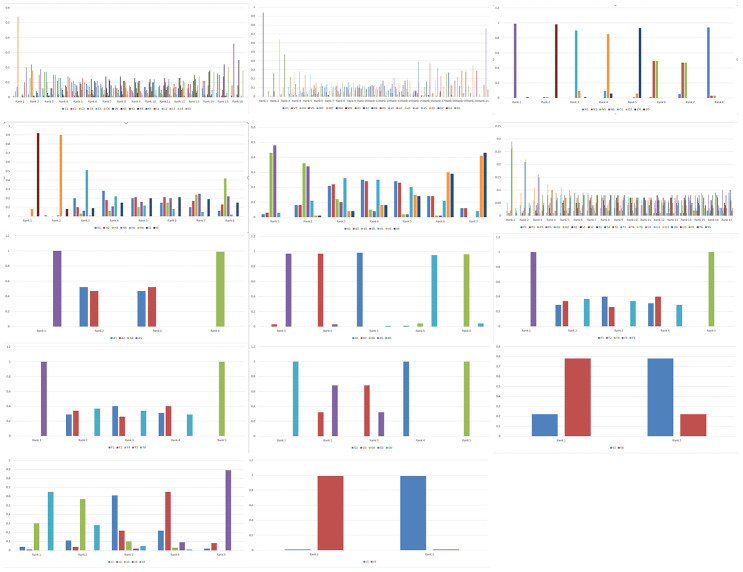
**Rank probabilities for the six genetic models of the SNPs related to PC risk.** The rank probabilities for the allele (1), homozygous (2), heterozygous (3), dominant (4), recessive (5) and additive (6) genetic models for the following SNPs: (C) *XPC* rs2607775; (D) *XPC* rs2228001; (K) *ERCC2* rs13181; (L) *ERCC1* rs3212986; (V) *ABO* rs657152; (W) *ABO* rs505922; (X) *ABO* rs495828; (V) *ABO* rs657152; (N) *COX2*-765; (O) *COX2*-1195; (H) *MUM1L1-CXorf57* rs379742; (I) *MORC4* rs12837024; (d) *HIF1α*-G1790A rs11549467; (e) *HIF1α*-C1772T rs11549465; (P) *CDKN2A/B* rs3731249; (Q) *CDKN2A/B* rs3731211; (R) *CDKN2A/B* rs3218009; (S) *CDKN2A/B* rs3217992; (T) *CDKN2A/B* rs2518719; (U) *CDKN2A/B* rs1063192; (Y) *CDKN2A/B* rs1063192; (A) *XRCC4* rs2075685; (B) *XRCC1* rs25487; (F) *VDR* rs2228570; (G) *TP53* rs9895829; (M) *CTLA-4* rs231775; (E) *VEGF* +405 rs2010963; (c) *MTHFR* rs1801133; (J) *FTO* rs9939609; (b) TERT rs2853677. Note: Genetic model of an SNP with best mean probability is considered the optimal genetic model.

We performed diagnostic meta-analysis of the additive gene model of *TP53* rs9895829 to evaluate the efficacy of this SNP to diagnose pancreatic cancer. As shown in Figure 2, the results of the diagnostic meta analysis for the additive gene model of *TP53* rs9895829 based on the random effects model were: DOR: 1.42 (95% CI, 1.27–1.59); pooled sensitivity: 0.55 (95% CI, 0.55 - 0.56); pooled specificity: 0.50 (95% CI, 0.48 - 0.52); +LR: 1.10 (95% CI, 1.06 - 1.14), and –LR: 0.90 (95% CI, 0.86 - 0.93).

## DISCUSSION

Several studies have investigated genetic susceptibility in PC, but the relationship between PC and SNPs is not conclusive. In this meta-analysis, we combined the results of several published studies to evaluate the association between PC and SNPs. We performed network meta-analysis, which is similar to pairwise meta-analysis, but is validated based on the quality of evidenceThe ranking probability was obtained by combining direct and indirect evidences with a Bayesian approach. A previous study successfully applied this approach to select the best genetic model for detecting the risk of hepatocellular carcinoma [61].

In our study, *TP53* rs9895829 showed stronger association with PC risk compared with *COX2*-765, *HIF-1α* rs11549467, *VDR* rs2228570, *TP53* rs9895829, *CTLA-4* rs231775, and *MTHFR* rs1801133. However, we were unable to conduct further subgroup analysis on TP53 rs9895829 to explore its specific association with PC because of smaller sample size.

In this study, we aimed to identify the most optimal genetic models among the six genetic models for the 30 SNPs that associate with PC risk by using network meta-analysis and Thakkinstian algorithm. Network meta-analysis is an extension to pairwise meta-analysis and, similar to pairwise meta-analysis, its validity is based on quality of evidence. The ranking probability was obtained from a combination of direct and indirect evidence with a Bayesian approach. dIn our study, *COX2*-765, *HIF-1α* rs11549467, *VDR* rs2228570, *TP53* rs9895829, *CTLA-4* rs231775, *MTHFR* rs1801133, *ABO* rs49582, *FTO* rs9939609, *CDKN2A/B* rs2518719, *XRCC4* rs2075685, *XRCC1* rs25487, *XPC* rs2607775, *MORC4* rs12837024, *VEGF* +405 rs2010963, and *MTHFR* rs1801133 were associated with greater risk of pancreatic cancer. The co-dominance model was the best genetic model to predict PC risk based on Thakkinstian's algorithm, but this model was not used in the pairwise meta-analysis (Table 2). We analyzed the correlation between PC and the genetic model based on the available data and did not consider any extrinsic factors that may affect the results in the study.

We then used FPRP values to determine the most plausible genetic model for the genes listed in Table 1. The three determinants of FPRP are prior probability, observed P value or α level, and statistical power, Wacholder et al. suggested that large studies or pooled analyses should use a stringent FPRP value below 0.2, prior probability as high (≈0.1), moderate (≈0.01), or low (≈0.001), and statistical power of 1.5 for alleles with higher cancer risk to obtain meaningful results [61, 62]. We chose moderate prior probability of 0.01 for FPRP and analyzed the 14 genes associated with pancreatic cancer. Our analysis showed that *TP53* rs9895829 was the best susceptibility gene for PC because it demonstrated a FPRP value of less than 0.2 even when the prior probability was 0.00001. The remaining 13 candidates showed FPRP values above 0.2 when prior probability value of 0.00001 was used.

Pancreatic cancer (PC) is a highly malignant cancer and is caused by a variety of factors of unknown etiology. The death rate of PC ranks eighth among all cancers worldwide and fourth among developed countries, with more than 260,000 deaths reported each year [63]. Most patients survive for less than a year after diagnosis and only 5% of PC patients survive for more than 5 years [64, 65]. The incidence of pancreatic cancer varies with population structure and the lifestyle of individuals [66, 67]. Identification of risk genes is critical in for decreasing the high mortality rates in PC. Our meta-analysis demonstrates the most relevant model for PC risk by collating the results of already published case-control studies related to PC. However, further high-quality studies with larger sample sizes and detailed PC risk factor data are necessary in the future to conclusively prove our findings.

Van et al. showed that vitamin D deficiency is very common in patients diagnosed with advanced pancreatic cancer [68]. Several studies have shown that 1, 25(OH)_2_Vitamin D regulates cellular proliferation, differentiation, apoptosis, and angiogenesis [64]. Colston et al. demonstrated that 1, 25(OH)_2_VitD and its synthetic analogues inhibited the proliferation of PC cell lines [69]. Vitamin D receptor (VDR) is expressed in the stroma of pancreatic tumors and mediates interstitial reprogramming to inhibit pancreatitis and pancreatic cancer [70]. VDR gene polymorphisms are associated with colon, breast, kidney, and prostate cancers [71–75]. Moreover, VDR gene polymorphisms affect immune response in immune-related diseases such as Graves' disease [76] and SLE [77]. The VDR rs2228570 T/C allele is 10 base pairs upstream of the translation initiation codon with the rs2228570 C allele variant generating shorter protein with higher activity than the rs2228570 T variant [78, 79]. Alimirah F et al. demonstrated that the T allele of rs2228570 increases breast tumor aggressiveness by up-regulating the expression of epidermal growth factor receptor (EGFR) [30, 80]. Li et al. showed that VDR rs2228570 gene polymorphism is associated with PC risk in the Northern China population [30]. *VDR* rs2228570 polymorphism also significantly correlates with pathological differentiation and TNM stages, and is a potential prognostic biomarker for PC [81].

The cytotoxic T-lymphocyte-associated protein 4 (*CTLA-4*) gene is located on chromosome 2q33 and encodes a crucial immune checkpoint protein on the T-lymphocytes; it consists of four exons that encode the leader sequence, the extracellular domain, the transmembrane domain, and the cytoplasmic domain [82]. Injection of anti-CTLA-4 antibody promotes anti-tumor immunity by enhancing the activation of T cells, thereby demonstrating its importance in tumorigenesis [83, 84]. The +49G>A allele in *CTLA-4* rs231775 changes of the amino acid from alanine^17^ to threonine^17^ and is associated with the high expression of CTLA-4, which inhibits T cell activation and proliferation [82, 85, 86]. In the Chinese population, the *CTLA-4* + 49a allele is associated with increased risk of lung, breast and cervical cancers [83, 85, 86]. Another meta-analysis showed that the *CTLA-4* +49A allele is associated with increased risk of pancreatic cancer in Caucasians and Chinese populations compared to the +49G allele [87]. In general, CTLA-4 is highly expressed in human pancreatic cancer cells [88]. The phase 2 trial of the anti-CTLA4 antibody, Ipilimumab, showed delayed progression in some advanced stage pancreatic cancer patients [89].

P53 protein encoded by the *TP53* gene plays a significant role in DNA damage, hypoxia, and metabolic stress, and inhibits tumorigenesis by regulating cell cycle and apoptosis [90]. Somatic mutations in the *TP53* gene have been reported in nearly 50% of human cancers, including pancreatic cancer [91, 92]. Morton et al. demonstrated that *TP53* mutations promote PC metastasis [93]. *TP53* gene mutations have been reported in 60% of sporadic pancreatic cancer cases and 33% of familial pancreatic cancer cases [94]. Biankin et al. demonstrated that *TP53* mutations are associated with susceptibility to pancreatic cancer [95]. Feng et al. showed that TP53 rs9895829 SNP was related to increased expression and activation of p53 in 373 lymphoblast cell lines [31].

Overall, our data suggests that several SNPs are potential candidates to diagnose PC because they are related to greater PC risk. However, because of smaller sample sizes and lack of sufficient information about extrinsic factors, we could not conduct sub-group analysis and optimal diagnostic meta-analysis of relevant indicators. Therefore, a single SNP may not be a sufficient indicator of PC risk, but, we postulate that analyzing multiple genes and SNPs may be a relevant diagnostic index for determining PC risk.

There are several limitations s in our study. Firstly, we lacked sufficient data to perform subgroup analysis and calculate heterogeneity. Secondly, we did not consider the potential impact of many extrinsic factors because these data were not available in the included studies. Thirdly, some of the included studies were of poor quality, which limited our ability to validate the combined results and perform subgroup analysis. Fourthly, it is plausible that because of our inclusion criteria, we excluded studies with relevant information about the SNPs. Hence, further analysis with large sample sizes and quality data is necessary to confirm our findings.

Nevertheless, this is the first systematic review and meta-analysis to our knowledge to comprehensively assess several SNPs associated with PC through network meta-analysis and Thakkinstian algorithm. We also measured the reliability of the meta-analysis results by FPRP to identify SNPs strongly associated with pancreatic cancer susceptibility. Our data suggests that some of the SNPs may be used in the future either alone or in combination for early screening of pancreatic cancer.

In conclusion, our data suggests that additive gene models of COX-2 -765, HIF-1α rs11549467, and TP53 rs9895829, as well as dominant gene models of DR rs2228570, CTLA-4 rs231775 and MTHFR rs1801133 are associated with PC risk. The additive genetic model for TP53 rs9895829 is the most optimal to diagnose PC risk. Future studies with large samples, detailed data on PC risk factors, and high-quality research are required to further validate the role of these PC risk-related SNPs.

## MATERIALS AND METHODS

This systematic review and meta-analysis was conducted in accordance with the guidelines and protocols of the systematic review and meta-analysis preferred reporting project (PRISMA) and registered in the INPLASY database (INPLASY202040023).

### Criteria for included studies

We included case-control studies on SNPs related to PC risk for this meta-analysis. We excluded repetitive reports, conference reports, review reports, news articles, animal studies, studies without sufficient data to calculate genotype distribution, and studies regarding SNPs that deviate from Hardy-Weinberg equilibrium (HWE). In these included studies, the experimental group included serum samples from PC patients that had not received any chemotherapy, whereas, the control group included healthy individuals, patients with non-malignant diseases, and non-cancer patients of different ages, gender, country, and tumor stage.

### Study search, selection and data extraction

We used terms such as single nucleotide polymorphism, SNP, pancreatic cancer and pancreatic tumor to search PubMed, Web of Science, Embase, Cochrane Library, China National Knowledge Infrastructure (CNKI), Science and Technology Periodical Database (VIP), and Wanfang databases for studies published until January 2020 without any language restrictions. The search criteria for the Pubmed database are shown in Supplementary Information 1.

**Figure 3:**
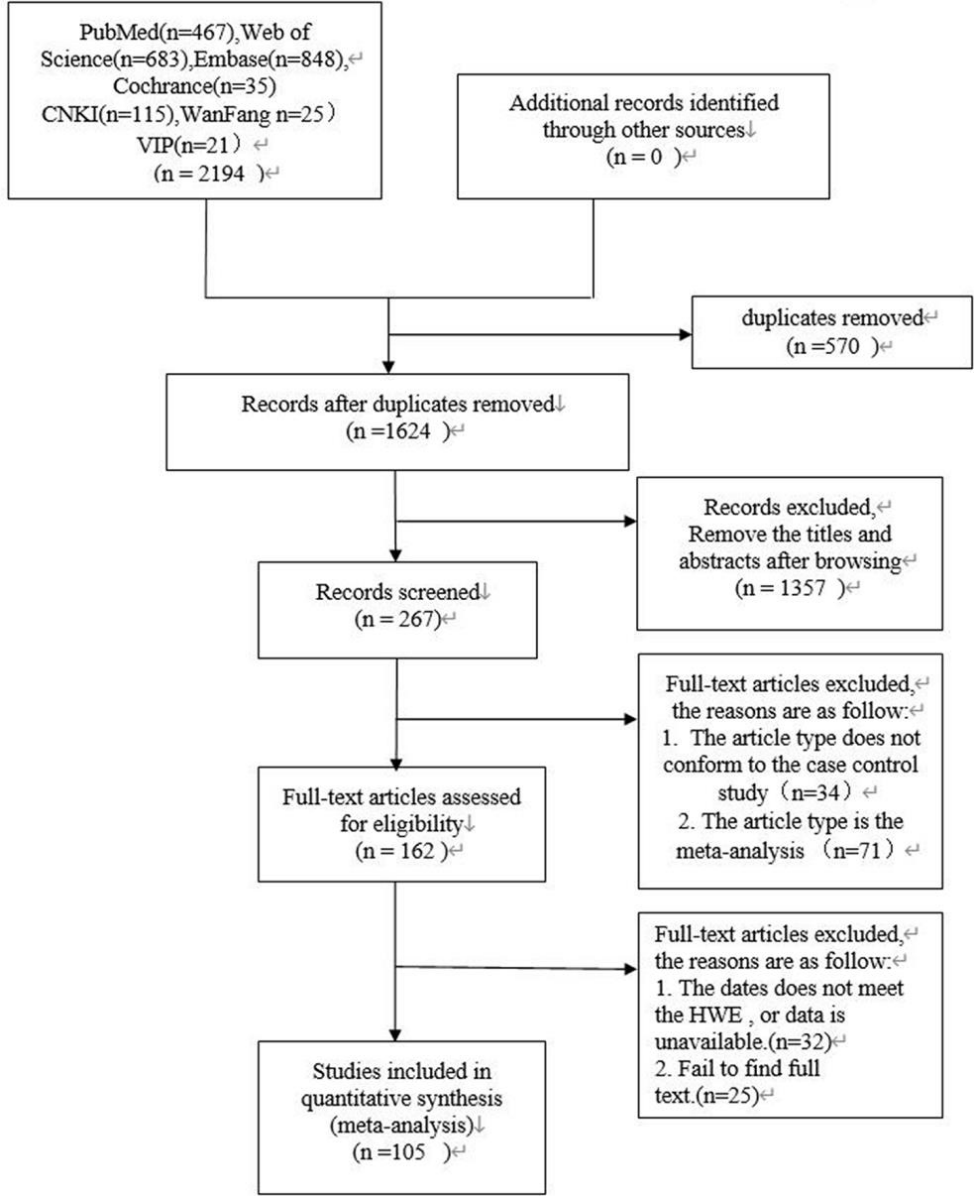
**PRISMA flow diagram of study search and selection.**

Data selection was performed independently by two reviewers (ZY and LL). In the case of disagreements, a third independent reviewer (JZ) was involved to reach consensus. The strategy used for study selection is shown in Figure 3. We extracted data including author names, year of publication, country, sample size of men and women, Hardy Weinberg equilibrium values, genotyping methods and genotype frequencies. The data was methodically evaluated by two independent reviewers (ZY and LL) according to the guidelines of the STREGA statement [23]. The third reviewer (JZ) was involved in resolving any issues between the two reviewers. The corresponding authors were contacted if any data was missing, insufficient, or vague. However, if relevant data was not obtained, those studies were excluded.

### Statistical analysis

The statistical data was analyzed using the StataMP14.0 software (https://www.stata.com/). The fixed effects or random effects pooled odds ratio (OR) were calculated using 95% confidence intervals (CIs) for pairwise meta-analysis based on the heterogeneity of the genetic models. We then conducted a network meta-analysis to determine the most suitable genetic model for each SNP.

The heterogeneity between studies was analyzed using I^2^ statistic and P value. Fixed effects model was used for studies with low heterogeneity as indicated by I^2^ value less than 50% and P value greater than 0.1. Otherwise, random effects model was used for studies with high heterogeneity. When sufficient data was available for SNPs with heterogeneity, we performed subgroup analysis to identify the source of heterogeneity and generate an optimal genetic model that can be used to predict PC susceptibility.

### Network meta-analysis

We used the ADDIS software (1.14) based on the Bayesian framework and Markov Chain Monte Carlo (MCMC) theory to generate mesh relationship diagram between genes related to PC risk. The four parallel Markov Chain Monte Carlo (MCMC) simulations underwent a burn-in phase of 20,000 stimulations and then an additional phase of 50,000 stimulations. The outcomes were evaluated by OR and 95% CI under random –effects model and consistency model was applied if 95% CI of log (OR) included 0. Otherwise, inconsistency model was used. The potential scale reduction factor (PSRF) was used to determine convergence. The model was considered convergent if the value of PSRF was closer to 1.0. This Bayesian method was used to rank each genetic model regarding the probability of PC risk and the corresponding rank probability map was automatically generated.

### Diagnostic meta-analysis

We performed diagnostic meta-analysis using the Meta-DiSc software [22] and evaluated sensitivity, specificity, likelihood ratios (LRs), diagnostic odds ratios (DORs), and summary receiver operating characteristic curves (SROC) of the SNPs to predict PC risk.

### False positive report probability (FPRP)

In the mesh meta-analysis, we used the Thakkinstian's algorithm [23] to evaluate the best genetic model for each SNP. A SNP consists of a dominant allele (G) and a recessive allele (g). Pairwise differences of GG vs. gg (D1), Gg vs. gg (D2), and GG vs. Gg (D3) were calculated as pooled OR1, OR2, and OR3, respectively, along with the corresponding 95% CIs. The most appropriate genetic model was determined to be recessive if OR1 = OR3 ≠ 1 and OR2 =1, dominant model if OR1 = OR2 ≠ 1 and OR3 =1, a complete over-dominant model if OR2 = 1/OR3 ≠1 and OR1 = 1, or codominant model if OR1 > OR2 > 1 and OR1 > OR3 > 1 (or if OR1 < OR2 < 1, and OR1 < OR3 < 1). We the FPRP by assuming a three-layer prior probability (low: 0.1; moderate: 0.01; high: 0.001) and an OR value of 1.5. The most suitable genetic model for each SNP related to PC risk was determined by comparing the results from network meta-analysis and the Thakkinstian algorithm [24].

## Supplementary Material

Supplementary Table 1

Supplementary Table 2

Supplementary Table 3

Supplementary Information 1

Supplementary Materials 1
