# Supplementary material for: A systematic review and network meta-analysis of single nucleotide polymorphisms associated with pancreatic cancer risk
Source: Aging (Albany NY). 2020 Nov 20;12(24):25256–74. doi: 10.18632/aging.104128 (PMC7803556; doi:10.18632/aging.104128)
Supplement: Supplementary Table 1 [file aging-12-104128-s001.pdf]

Supplementary Table 1. The methodological quality evaluation results were included.

	STREGA evaluation index									
**Included studies**	①	②	③	④	⑤	⑥	⑦	⑧	⑨	**Total pionts**
Cosmeri Rizzato	1	0	0	1	0	1	1	0	1	5
COSMERI RIZZATO	1	1	0	1	0	1	1	0	1	6
Cuicui Lang	1	1	0	1	0	1	1	0	1	6
DAN ZHAO	1	1	0	1	0	1	1	0	1	6
Daniele Campa 2015	1	1	0	1	0	1	1	0	1	6
Daniele Campa 2016	1	1	0	1	0	1	1	0	1	6
Daniele Campa 2017	1	1	0	1	0	1	1	0	1	6
Daniele Campa 2018	1	0	0	1	0	1	1	0	0	4
Dimitrios Karakaxas	1	1	0	1	0	1	1	0	1	6
Donghui Li	1	1	0	1	0	1	1	0	1	6
Fuli Zhao	1	1	0	1	0	1	1	0	1	6
GUO-YANG WU	1	1	0	1	0	1	1	0	1	6
Hideo Suzuki	1	1	0	1	0	1	1	0	1	6
Hong-Li Xu	1	1	0	1	0	1	1	0	1	6
Hongwei Tang	1	1	0	1	0	1	1	0	1	6
Ivan Nisevic	1	0	0	1	0	1	1	0	1	5
L.J. Wang	1	1	0	1	0	1	1	0	1	6
Lei Li	1	1	0	1	0	1	1	0	1	6
LEI LI	1	0	0	1	0	1	1	0	0	4
Lei Zhao	1	1	1	1	0	1	1	0	1	7
LI WANG	1	1	0	1	0	1	1	0	1	6
Liu, Chengli	1	1	0	1	0	1	1	0	1	6
M.F. Ying	1	0	0	1	0	1	1	0	0	4
Makoto Nakao 2011	1	1	0	1	0	1	1	0	1	6
Makoto Nakao 2012	1	1	1	1	0	1	1	0	1	7
Ming Yang	1	1	0	1	0	1	1	0	1	6
Moschovis, D	1	1	0	1	0	1	1	0	1	6
Ofure Obazee	1	0	0	1	0	1	1	0	0	4
Pinghai Hu	1	1	1	1	0	1	1	0	1	7
Qicai Liu	1	1	0	1	0	1	1	0	1	6
Quan Shen	1	1	0	1	0	1	1	0	1	6
R. TALAR-WOJNAROWSKA2010	1	1	0	1	0	1	1	0	1	6
R. TALAR-WOJNAROWSKA2009	1	1	0	1	0	0	1	0	0	4
Ruiz-Tovar, J	1	0	0	1	0	1	1	0	0	4
Siddapuram Sivaprasad	1	1	1	1	0	1	1	0	1	7
XIAO-HUI LIANG	1	1	0	1	0	1	1	0	1	6
Xinyuan, Xu	1	0	1	1	0	1	1	0	0	5
Xiuchao Wang	1	1	0	1	0	1	1	0	1	6
Y. Ding	1	1	0	1	0	1	1	0	1	6
Yang Fei	1	0	0	1	0	1	1	0	1	5
Yingsong Lin	1	1	0	1	0	1	1	0	1	6
Yun Feng	1	0	1	1	0	1	1	0	0	5
Tieying He	1	0	0	1	0	1	1	0	0	4
Dongkui Xu	1	0	0	1	0	1	1	0	0	4
Dong Yan	1	0	0	1	0	1	1	0	0	4

Note: ①whether genotyping methods described, ②whether to describe population stratification method, ③whether to describe genotype inference method, ④control group genotype distribution is in line with the hardy-weinberg balance law, ⑤whether the emphasis on study of repetitive, ⑥whether to describe the object of study, exclusion criteria and matching method, ⑦whether to show statistical methods and software versions, ⑧relevance judgment method, ⑨whether the data is sufficient.
