# Supplementary material for: A systematic review and network meta-analysis of single nucleotide polymorphisms associated with pancreatic cancer risk
Source: Aging (Albany NY). 2020 Nov 20;12(24):25256–74. doi: 10.18632/aging.104128 (PMC7803556; doi:10.18632/aging.104128)
Supplement: Supplementary Table 2 [file aging-12-104128-s002.docx]

**Supplementary Table 2. Characteristics of studies included for meta-analysis.**

| First author, year of publication[reference] | Country | Studied SNPs | Genotyping method | Sample size(case/control) | P_HWE_ |
| --- | --- | --- | --- | --- | --- |
| Y. Ding 2015 | China | XRCC4 rs2075685,XRCC4 rs1805377 | PCR-RFLP | 206/412 | 0.2654/0.2079 |
| Quan Shen 2015 | China | XRCC4 rs2075685,XRCC4 rs1805377 | PCR-RFLP | 248/496 | 0.0982/0.1043 |
| Makoto Nakao 2012 | Japan | XRCC1 rs25487,XRCC1 rs1799782,OGG1 rs1052133 | TaqMan | 185/1465 | 0.9386/0.8842/0.0400 |
| Dong Yan 2012 | China | XRCC1 rs25487,XRCC1 rs1799782 | RCR | 210/213 | 0.4579/0.4579 |
| XIAO-HUI LIANG 2018 | China | XPC rs2607775,XPC rs2228001,  XPC rs3731055,XPC rs2228000 | PCR | 205/230 | 0.1485/0.6331/  0.3476/0.3001 |
| Tieying He 2013 | China | XPC rs2607775, XPC rs3731055 | PCR | 210/214 | 0.3045/0.5039 |
| R.TALAR-WOJNAROWSKA 2010 | Poland | VEGF +405 rs2010963,VEGT -460 rs833061 | PCR | 85/50 | 0.1419/0.6631 |
| Siddapuram Sivaprasad 2013 | India | VEGF +405 rs2010963,VEGT -460 rs833061 | PCR–RFLPs | 80/87 | 0.0775/0.0058 |
| Lei Li 2015 | China | VDR rs2228570,VDR rs1544410 | PCR-RFLP | 258/385 | 0.0160/0.4212 |
| LEI LI 2013 | China | VDR rs2228570,VDR rs1544410 | PCR | 91/80 | 0.2069/0.6367 |
| Yun Feng 2019 | Europe | TP53 rs9895829 | eQTL | 8477/6946 | 0.4349 |
| Xinyuan, Xu 2019 | Europe | TP53 rs9895829 | PCR | 8474/6944 | 0.4790 |
| Yingsong Lin 2013 | Japan | FTO rs9939609 | TaqMan | 360/400 | 0.8917 |
| Hongwei Tang 2011 | America | FTO rs9939609a | TaqMan | 1070/1174 | 0.7060 |
| Fuli Zhao 2015 | China | XPC rs2228001, ERCC2 rs13181,ERCC1 rs3212986,  XPC rs2228000,ERCC4 rs6498486,ERCC1 rs11615 | PCR-RFLP | 246/246 | 0.1263/<0.001/0.2138/  0.0081/0.1803/0.9371 |
| M.F. Ying 2016 | China | ERCC2 rs13181,ERCC1 rs3212986,  ERCC1 rs11615,ERCC4 rs6498486 | PCR-RFLP | 217/272 | 0.0002/0.4905/  0.3612/0.5704 |
| Ming Yang, PhD 2012 | China | CTLA-4 rs231775 | PCR | 368/926 | 0.0063 |
| Cuicui Lang 2011 | China | CTLA-4 rs231775 | PCR | 602/651 | 0.8278 |
| DAN ZHAO 2009 | China | COX-2-765,COX-2-1195,COX-2-1290 | PCR | 393/786 | 0.5854/0.5213/0.3472 |
| Dongkui Xu 2008 | China | COX-2-765,COX-2-1195,COX-2-1290 | PCR | 283/566 | 0.6063/0.8930/0.3360 |
| Daniele Campa 2016(A) | Germany  Czech republic  Greece  Italy  Lithuania  Poland  Netherlands  UnitedKingdom | CDKN2A/Brs3731249,CDKN2A/Brs3731257,  CDKN2A/Brs3731239,CDKN2A/Brs3217986,  CDKN2A/B rs2811710,CDKN2A/B rs11515,  CDKN2A/Brs3731211,CDKN2A/B 3218009,  CDKN2A/Brs3217992,CDKN2A/Brs2518719,  CDKN2A/B rs1063192,CDKN2A/B rs2811708 | TaqMan,KASPar | 2712/5422 | 0.4782/0.1546/  0.21980.1950/  0.3845/0.9918/  0.0369/0.0276/  0.5968/0.1267/  0.5720/0.8951 |
| Daniele Campa 2016(B) | Germany  Czech republic  Greece  Italy  Lithuania  Poland  Netherlands  United Kingdom | CDKN2A/Brs3731249,CDKN2A/Brs3731257,  CDKN2A/Brs3731239,CDKN2A/Brs3217986,  CDKN2A/B rs2811710,CDKN2A/B rs11515,  CDKN2A/Brs3731211,CDKN2A/B 3218009,  CDKN2A/Brs3217992,CDKN2A/Brs2518719,  CDKN2A/B rs1063192,CDKN2A/B rs2811708 | TaqMan,KASPar | 145/689 | <0.0001/0.638/  0.771/0.143/  0.143/1.000/  0.215/<0.0001/  0.075/<0.0001/  0.142/0.796 |
| Hong-Li Xu 2014 | China | ABO rs657152,ABO rs505922 ABO rs495828 | TaqMan | 230/458 | 0.0226/0.0465/0.1857 |
| Cosmeri Rizzato 2011 | Germany  United Kingdom | ABO rs657152,ABO rs505922,ABO rs495828,TERT rs401681 | PCR-based KASPar | 690/1277 | 0.2715/0.5810/  0.3672/0.9494 |
| Ofure Obazee 2018 | Germany  Greece  Italy  Poland  United Kingdom | ABO rs505922,TERT rs2736098,  XTERT rs401681, TERT rs2853677 | TaqMan | 369/3277 | 0.0419/0.9911/  0.3645/0.5830 |
| COSMERI RIZZATO 2013 | Germany  Czech republic  Italy  Poland | ABO rs505922 | PCR-based KASPar | 1028/2257 | 0.6717 |
| Daniele Campa 2015 | Europe | TERT rs2736098,TERT rs401681,TERT rs2853677 | TaqMan | 1091/4106 | 0.3721/0.4819/0.1299 |
| Daniele Campa 2018(A) | Germany  Greece  Italy  Poland  United Kingdom | SAMD12-TNFRSF11B rs11988997,  PRSS1-PRSS2 rs10273639,  KIAA1462-MTPAP rs2995271,  UM1L1-CXorf57 rs379742,  MORC4 rs12837024 | Human 660w-quad array | 2754/4890 | 0.6889  0.1108  0.1576  0.0559  <0.0001 |
| Daniele Campa 2018(B) | Japan | SAMD12-TNFRSF11B rs11988997,  PRSS1-PRSS2 rs10273639,  KIAA1462-MTPAP rs2995271,  UM1L1-CXorf57 rs379742,  MORC4 rs12837024 | Human 660w-quad array | 160/706 | 0.7245  0.2752  0.8990  <0.0001  <0.0001 |
| Qicai Liu 2011 | China | PRSS1 rs10273639 | PCR | 154/520 | 0.8445 |
| Donghui Li2009 | America | OGG1 rs1052133 | TaqMan Masscode | 734/780 | 0.8864 |
| Moschovis, D 2019 | Greece | HOTAIR rs4759314 | PCR-RFLP | 111/125 | 0.7355 |
| Pinghai Hu 2017 | China | HOTAIR rs4759314 | TaqMan | 416/416 | 0.1656 |
| Yang Fei 2010 | China | E-cadherine -160 rs16260 | PCR | 254/101 | 0.5052 |
| Lei Zhao 2015 | China | E-cadherine -160 rs16260 | PCR | 368/376 | 0.8722 |
| GUO-YANG  WU 2010 | Germany | TNF-α-308 rs1800629 | PCR | 73/117 | 0.7153 |
| R.TALAR-WOJNAROWSKA2009 | Poland | TNF-α rs1800629 | RFLP-PCR | 41/50 | 0.8613 |
| Dimitrios Karakaxas 2014 | Greece | TNF-a-308 rs1800629 | PCR-RFLP | 78/98 | 0.7133 |
| Daniele Campa 2017 | Germany  Italy  Poland  United Kingdom | TNFA rs1800629 | KBiosciences | 344/2721 | 0.2417 |
| Liu, Chengli2014 | China | CLPTM1L-TERT rs401681 | TaqMan assay | 766/821 | 0.2631 |
| LI WANG 2005 | China | MTHFR-677 rs1801133 | PCR | 163/337 | 0.2701 |
| Ivan Nisevic 2008 |  | MTHFR rs1801133 | PCR | 21/50 | 0.1912 |
| Makoto Naka 2011 | Japan | IGF-1 rs2288378,IGF-1 rs5742714 | TaqMan | 176/1402 | 0.0623/0.1330 |
| Hideo Suzuki 2008 | America | IGF-1 rs2288378,IGF-1-177 rs5742714 | TaqMan | 892/783 | 0.5420/0.8570 |
| Ruiz-Tovar, J 2012 | Spain | HIF-1α-G1790A rs11549467,  HIF-1α-C1772T rs11549465 | PCR-RFLP | 59/159 | 0.6750  0.0016 |
| Xiuchao Wang 2011 | China | HIF-1α rs11549467,HIF-1α rs11549465 | PCR | 263/271 | 0.4861/0.3521 |

Note: 1.P_HWE_ represents the P value of hardy-weinberg balance law test in the control group in the study.

2.Daniele Campa 2016 and Daniele Campa 2018 studied different Numbers of people in two regions, Europe and Japan, so they were separated as a separate data(A and B).
