# Supplementary material for: A systematic review and network meta-analysis of single nucleotide polymorphisms associated with pancreatic cancer risk
Source: Aging (Albany NY). 2020 Nov 20;12(24):25256–74. doi: 10.18632/aging.104128 (PMC7803556; doi:10.18632/aging.104128)
Supplement: Supplementary Table 3 [file aging-12-104128-s003.docx]

**Supplementary Table 3. Pairwise meta-analysis of the selected SNPs in association with risk for pancreatic cancer(PC).**

|  | Sample size | | Heterogeneity | |  |  |
| --- | --- | --- | --- | --- | --- | --- |
| Genetic model | Case/control | | I² | P | Model | OR(95%CI) |
| XRCC4 rs2075685 | | 454/908 |  |  |  |  |
| GvsT* |  | | 0.00% | 0.655 | Fixed | 0.746(0.636-0.876) |
| GGvsTT* |  | | 0.00% | 0.645 | Fixed | 0.578(0.423-0.790) |
| GTvsTT |  | | 0.00% | 0.74 | Fixed | 0.781(0.589-1.036) |
| GGvsGT+TT* |  | | 0.00% | 0.755 | Fixed | 0.681(0.530-0.875) |
| GG+GTvsTT* |  | | 0.00% | 0.668 | Fixed | 0.695(0.534-0.904) |
| GG+TTvsGT |  | | 0.00% | 0.978 | Fixed | 0.940(0.750-1.178) |
| XRCC4 rs1805377 | 454/908 | |  |  |  |  |
| GvsA |  | | 0.00% | 0.86 | Fixed | 1.102(0.936-1.296) |
| GGvsAA |  | | 0.00% | 0.871 | Fixed | 1.187(0.855-1.647) |
| GAvsAA |  | | 0.00% | 0.935 | Fixed | 1.122(0.875-1.439) |
| GGvsGA+AA |  | | 0.00% | 0.883 | Fixed | 1.115(0.828-1.502) |
| GG+GAvsAA |  | | 0.00% | 0.9 | Fixed | 1.140(0.903-1.438) |
| GG+AAvsGA |  | | 0.00% | 0.977 | Fixed | 0.94(0.749-1.178） |
| XRCC1 rs25487 | 395/1678 | |  |  |  |  |
| GvsA* |  | | 0.00% | 0.329 | Fixed | 0.726(0.601-0.887) |
| GGvsAA* |  | | 0.00% | 0.499 | Fixed | 0.545(0.345-0.863) |
| GAvsAA |  | | 0.00% | 0.777 | Fixed | 0.767（0.479-1.230） |
| GGvsGA+AA* |  | | 0.00% | 0.389 | Fixed | 0.688(0.541-0.874) |
| GG+GAvsAA* |  | | 0.00% | 0.581 | Fixed | 0.627(0.401-0.980) |
| GG+AAvsGA* |  | | 0.00% | 0.575 | Fixed | 0.770(0.603-0.984） |
| XRCC1 rs1799782 | 395/1678 | |  |  |  |  |
| TvsC |  | | 84.20% | 0.012 | Random | 1.197(0.734-1.949) |
| TTvsCC |  | | 74.80% | 0.046 | Random | 1.332(0.528-3.365) |
| TCvsCC |  | | 69.40% | 0.07 | Random | 1.209(0.756-1.932) |
| TTvsTC+CC |  | | 62.60% | 0.102 | Random | 1.218(0.584-2.541) |
| TT+TCvsCC |  | | 79.90% | 0.026 | Random | 1.238(0.714-2.146) |
| TT+CCvsTC |  | | 48.20% | 0.165 | Fixed | 0.880(0.691-1.122) |
| XPC rs3731055 | 415/444 | |  |  |  |  |
| AvsG |  | | 0.00% | 0.626 | Fixed | 1.002(0.801-1.252) |
| AAvsGG |  | | 0.00% | 0.599 | Fixed | 0.963(0.551-1.681) |
| AGvsGG |  | | 0.00% | 0.84 | Fixed | 1.024（0.768-1.364） |
| AAvsAG+GG |  | | 0.00% | 0.619 | Fixed | 0.954（0.552-1.650） |
| AA+AGvsGG |  | | 0.00% | 0.728 | Fixed | 1.014(0.772-1.332) |
| AA+GGvsAG |  | | 0.00% | 0.917 | Fixed | 0.973（0.734-1.290） |
| XPC rs2607775 | 415/444 | |  |  |  |  |
| GvsC* |  | | 0.00% | 0.848 | Fixed | 1.895(1.389-2.586) |
| GGvsCC* |  | | 0.00% | 0.388 | Fixed | 3.523（1.176-10.551） |
| GCvsCC* |  | | 0.00% | 0.876 | Fixed | 1.797（1.263-2.555） |
| GGvsGC+CC* |  | | 0.00% | 0.377 | Fixed | 3.123（1.048-9.313） |
| GG+GCvsCC* |  | | 0.00% | 0.983 | Fixed | 1.913（1.361-2.690） |
| GG+CCvsGC* |  | | 0.00% | 0.894 | Fixed | 0.573（0.403-0.814） |
| XPC rs2228001 | 451/476 | |  |  |  |  |
| AvsC |  | | 93.90% | <0.001 | Random | 1.559(0.741-3.281) |
| AAvsCC |  | | 94.10% | <0.001 | Random | 2.421（0.465-12.595） |
| ACvsCC |  | | 95.70% | <0.001 | Random | 2.348（0.344-16.018） |
| AAvsAC+CC* |  | | 49.50% | 0.16 | Random | 1.413（1.101-1.814） |
| AA+ACvsCC |  | | 95.60% | <0.001 | Random | 2.374（0.396-14.232） |
| AA+CCvsAC |  | | 89.40% | 0.002 | Random | 0.783（0.359-1.708） |
| XPC rs2228000 | 451/476 | |  |  |  |  |
| TvsC |  | | 0.00% | 0.588 | Fixed | 1.059(0.923-1.214) |
| TTvsCC |  | | 0.00% | 0.528 | Fixed | 1.047（0.683-1.603） |
| TCvsCC |  | | 0.00% | 0.86 | Fixed | 1.210（0.911-1.583） |
| TTvsTC+CC |  | | 0.00% | 0.534 | Fixed | 0.966（0.642-1.454） |
| TT+TCvsCC |  | | 0.00% | 0.733 | Fixed | 1.165（0.900-1.508） |
| TT+CCvsTC |  | | 0.00% | 0.995 | Fixed | 0.839（0.643-1.093） |
| VEGF -460 rs833061 | 165/137 | |  |  |  |  |
| TvsC |  | | 2.50% | 0.311 | Fixed | 1.145（0.827-1.584） |
| TTvsCC |  | | 19.20% | 0.266 | Fixed | 1.254（0.641-2.455） |
| TCvsCC |  | | 51.40% | 0.152 | Random | 0.780（0.336-1.807） |
| TTvsTC+CC |  | | 0.00% | 0.797 | Fixed | 1.535（0.897-2.625） |
| TT+TCvsCC |  | | 55.80% | 0.133 | Random | 0.920（0.400-2.116） |
| TT+CCvsTC |  | | 5.40% | 0.304 | Fixed | 1.456（0.917-2.313） |
| VEGF +405 rs2010963 | 165/137 | |  |  |  |  |
| GvsC |  | | 93.60% | <0.001 | Random | 0.734（0.189-2.857） |
| GGvsCC |  | | 85.00% | 0.01 | Random | 0.495（0.073-3.366） |
| GCvsCC* |  | | 70.70% | 0.065 | Random | 0.265（0.075-0.937） |
| GGvsGC+CC |  | | 83.50% | 0.014 | Random | 1.166（0.310-4.379） |
| GG+GCvsCC |  | | 84.20% | 0.012 | Random | 0.348（0.069-1.770） |
| GG+CCvsGC* |  | | 0.00% | 0.407 | Fixed | 2.838（1.765-4.563） |
| VDR rs2228570 | 349/465 | |  |  |  |  |
| CvsT* |  | | 0.00% | 0.864 | Fixed | 0.530（0.433-0.649） |
| CCvsTT* |  | | 0.00% | 0.899 | Fixed | 0.326（0.218-0.488） |
| CTvsTT |  | | 0.00% | 0.897 | Fixed | 0.737（0.504-1.076） |
| CCvsCT+TT* |  | | 0.00% | 0.775 | Fixed | 0.403（0.297-0.547） |
| CC+CTvsTT* |  | | 0.00% | 0.98 | Fixed | 0.571（0.363-0.737） |
| CC+TTvsTC* |  | | 0.00% | 0.787 | Fixed | 0.667（0.503-0.884） |
| VDR rs1544410 | 349/465 | |  |  |  |  |
| AvsG |  | | 88.20% | 0.004 | Random | 0.947（0.469-1.910） |
| AAvsGG |  | | 87.50% | 0.005 | Random | 0.873（0.220-3.465） |
| AGvsGG |  | | 72.70% | 0.056 | Random | 0.749（0.321-1.749） |
| AAvsAG+GG |  | | 78.00% | 0.033 | Random | 1.112（0.504-2.451） |
| AA+AGvsGG |  | | 84.10% | 0.012 | Random | 0.785（0.273-2.256） |
| AA+GGvsAG |  | | 0.00% | 0.864 | Fixed | 1.279(0.967-1.692) |
| TP53 rs9895829 | 16951/13890 | |  |  |  |  |
| GvsA* |  | | 0.00% | 0.901 | Fixed | 0.815（0.759-0.874） |
| GGvsAA |  | | 0.00% | 0.953 | Fixed | 0.691（0.440-1.086） |
| GAvsAA* |  | | 0.00% | 0.909 | Fixed | 0.811（0.754-0.874） |
| GGvsGA+AA* |  | | 0.00% | 0.954 | Fixed | 0.706(0.449-1.109) |
| GG+GAvsAA |  | | 0.00% | 0.904 | Fixed | 0.808(0.751-0.870) |
| GG+AAvsGA* |  | | 0.00% | 0.909 | Fixed | 1.231(1.143-1.326) |
| TERT rs2736098 | 1460/7383 | |  |  |  |  |
| AvsG |  | | 2.00% | 0.313 | Fixed | 0.926（0.851-1.007） |
| AAvsGG |  | | 0.00% | 0.753 | Fixed | 0.927（0.754-1.140） |
| AGvsGG |  | | 73.60% | 0.051 | Random | 0.941（0.723-1.225） |
| AAvsAG+GG |  | | 0.00% | 0.463 | Fixed | 0.973（0.796-1.191) |
| AA+AGvsGG |  | | 62.10% | 0.104 | Random | 0.930（0.755-1.146） |
| AA+GGvsAG |  | | 76.60% | 0.039 | Random | 1.048（0.797-1.379） |
| SAMD12-TNFRSF11B rs11988997 | 6330/11574 | |  |  |  |  |
| TvsC |  | | 0.00% | 0.819 | Fixed | 0.889（0.786-1.007） |
| TTvsCC |  | | 62.10% | 0.105 | Random | 0.961（0.309-2.985） |
| TCvsCC |  | | 0.00% | 0.546 | Fixed | 0.889（0.778-1.016） |
| TTvsTC+CC |  | | 64.10% | 0.095 | Random | 0.993（0.310-3.182） |
| TT+TCvsCC |  | | 0.00% | 0.831 | Fixed | 0.885（0.776-1.009） |
| TT+CCvsTC |  | | 0.00% | 0.502 | Fixed | 1.123（0.983-1.283） |
| PRSS1-PRSS2 rs10273639 | 6484/12094 | |  |  |  |  |
| CvsT |  | | 84.50% | 0.002 | Random | 0.957(0.895-1.023) |
| CCvsTT |  | | 39.00% | 0.2 | Fixed | 0.901（0.787-1.032） |
| CTvsTT |  | | 0.00% | 0.457 | Fixed | 0.942（0.832-1.065） |
| CCvsCT+TT |  | | 87.30% | 0 | Random | 0.960(0.869-1.061) |
| CC+CTvsTT |  | | 0.00% | 0.907 | Fixed | 0.924（0.823-1.038) |
| CC+TTvsTC |  | | 86.40% | 0.001 | Random | 1.015(0.926-1.114) |
| OGG1 rs1052133 | 919/2245 | |  |  |  |  |
| GvsC |  | | 0.00% | 0.502 | Fixed | 1.012（0.885-1.157） |
| GGvsCC |  | | 0.00% | 0.953 | Fixed | 0.908（0.662-1.246） |
| GCvsCC |  | | 0.00% | 0.408 | Fixed | 1.086（0.903-1.305） |
| GGvsGC+CC |  | | 0.00% | 0.746 | Fixed | 0.910（0.683-1.212） |
| GG+GCvsCC |  | | 0.00% | 0.42 | Fixed | 1.058（0.887-1.261） |
| GG+CCvsGC |  | | 0.00% | 0.445 | Fixed | 0.914(0.768-1.087) |
| MUM1L1-CXorf57 rs379742 | 6330/11574 | |  |  |  |  |
| AvsG* |  | | 99.50% | 0.000 | Random | 0.226（0.206-0.249） |
| AAvsGG* |  | | 99.30% | 0.000 | Random | 0.161（0.135-0.191） |
| AGvsGG* |  | | 98.40% | 0.000 | Random | 0.101（0.085-0.121） |
| AAvsAG+GG* |  | | 98.40% | 0.000 | Random | 0.329（0.284-0.381） |
| AA+AGvsGG* |  | | 99.40% | 0.000 | Random | 0.132（0.115-0.152） |
| AA+GGvsAG* |  | | 95.90% | 0.000 | Random | 4.720（4.071-5.471） |
| MORC4 rs12837024 | 6330/11574 | |  |  |  |  |
| TvsC |  | | 0.00% | 0.608 | Fixed | 1.009（0.933-1.092） |
| TTvsCC |  | | 0.00% | 0.942 | Fixed | 1.064（0.934-1.212） |
| TCvsCC* |  | | 48.90% | 0.162 | Fixed | 0.860（0.751-0.986） |
| TTvsTC+CC |  | | 0.00% | 0.751 | Fixed | 1.092（0.961-1.240） |
| TT+TCvsCC |  | | 0.00% | 0.368 | Fixed | 0.959（0.865-1.063） |
| TT+CCvsTC* |  | | 49.20% | 0.161 | Fixed | 1.176（1.029-1.344） |
| KIAA1462-MTPAP rs2995271 | 6330/11574 | |  |  |  |  |
| TvsC |  | | 0.00% | 0.683 | Fixed | 0.977（0.902-1.058） |
| TTvsCC |  | | 0.00% | 0.994 | Fixed | 1.042（0.838-1.298） |
| TCvsCC |  | | 32.50% | 0.223 | Fixed | 1.013（0.829-1.238） |
| TTvsTC+CC |  | | 0.00% | 0.670 | Fixed | 0.966（0.876-1.065） |
| TT+TCvsCC |  | | 0.00% | 0.333 | Fixed | 0.999（0.825-1.211） |
| TT+CCvsTC |  | | 34.40% | 0.217 | Fixed | 0.966（0.875-1.066） |
| HOTAIR rs4759314 | 527/541 | |  |  |  |  |
| GvsA |  | | 70.60% | 0.065 | Random | 1.033（0.794-1.343） |
| GGvsAA |  | | 62.80% | 0.101 | Random | 1.339（0.585-3.066） |
| GAvsAA |  | | 0.00% | 0.322 | Fixed | 0.972（0.717-1.318） |
| GGvsGA+AA |  | | 59.90% | 0.114 | Random | 1.345（0.587-3.080） |
| GG+GAvsAA |  | | 51.90% | 0.149 | Random | 1.003（0.749-1.343） |
| GG+AAvsGA |  | | 0.00% | 0.396 | Fixed | 1.038（0.766-1.407） |
| FTO rs9939609 | 1430/1575 | |  |  |  |  |
| AvsT* |  | | 54.30% | 0.139 | Random | 1.124（1.008-1.254） |
| AAvsTT |  | | 0.00% | 0.715 | Fixed | 1.194（0.939-1.518） |
| ATvsTT |  | | 67.90% | 0.077 | Random | 1.151（0.983-1.347） |
| AAvsAT+TT |  | | 0.00% | 0.896 | Fixed | 1.147（0.919-1.431） |
| AA+ATvsTT* |  | | 63.20% | 0.099 | Random | 1.168（1.006-1.357） |
| AA+TTvsAT |  | | 74.80% | 0.046 | Random | 0.914（0.789-1.058） |
| ERCC4 rs6498486 | 463/518 | |  |  |  |  |
| CvsA |  | | 0.00% | 0.855 | Fixed | 1.146（0.936-1.404） |
| CCvsAA |  | | 0.00% | 0.719 | Fixed | 1.338（0.830-2.156） |
| CAvsAA |  | | 0.00% | 0.964 | Fixed | 1.118（0.852-1.468） |
| CCvsCA+AA |  | | 0.00% | 0.721 | Fixed | 1.276（0.803-2.027） |
| CC+CAvsAA |  | | 0.00% | 0.915 | Fixed | 1.154（0.893-1.493） |
| CC+AAvsCA |  | | 0.00% | 0.986 | Fixed | 0.931（0.715-1.212） |
| ERCC2 rs13181 | 434/516 | |  |  |  |  |
| GvsT* |  | | 44.00% | 0.181 | Fixed | 1.440（1.171-1.770） |
| GGvsTT* |  | | 9.50% | 0.293 | Fixed | 1.860（1.240-2.790） |
| GTvsTT |  | | 0.00% | 0.521 | Fixed | 1.245（0.927-1.671） |
| GGvsGT+TT* |  | | 0.00% | 0.354 | Fixed | 1.733（1.168-2.571） |
| GG+GTvsTT* |  | | 7.10% | 0.299 | Fixed | 1.406（1.082-1.828） |
| GG+TTvsGT |  | | 0.00% | 0.719 | Fixed | 0.894（0.671-1.190） |
| ERCC1 rs3212986 | 434/516 | |  |  |  |  |
| TvsG* |  | | 0.00% | 0.985 | Fixed | 1.462（1.213-1.763） |
| TTvsGG* |  | | 0.00% | 0.888 | Fixed | 2.328（1.540-3.517） |
| TGvsGG* |  | | 0.00% | 0.994 | Fixed | 1.381（1.042-1.829） |
| TTvsTG+GG* |  | | 0.00% | 0.875 | Fixed | 1.936（1.325-2.830） |
| TT+TGvsGG* |  | | 0.00% | 0.995 | Fixed | 1.547（1.183-2.021） |
| TT+GGvsTG |  | | 0.00% | 0.977 | Fixed | 0.910（0.704-1.177） |
| ERCC1 rs11615 | 463/518 | |  |  |  |  |
| CvsT |  | | 0.00% | 0.957 | Fixed | 0.881（0.724-1.072） |
| CCvsTT |  | | 0.00% | 0.994 | Fixed | 0.755（0.473-1.206） |
| CTvsTT |  | | 0.00% | 0.998 | Fixed | 0.847（0.528-1.356） |
| CCvsCT+TT |  | | 0.00% | 0.974 | Fixed | 0.867（0.670-1.121） |
| CC+CTvsTT |  | | 0.00% | 0.996 | Fixed | 0.797（0.509-1.251） |
| CC+TTvsTC |  | | 0.00% | 0.977 | Fixed | 0.933（0.720-1.208） |
| E-cadherine -160 rs16260 | 622/477 | |  |  |  |  |
| AvsC |  | | 91.10% | 0.001 | Random | 0.942(0.789-1.125) |
| AAvsCC |  | | 91.90% | 0.000 | Random | 0.861（0.588-1.260） |
| ACvsCC |  | | 58.90% | 0.119 | Random | 0.994(0.763-1.294) |
| AAvsAC+CC |  | | 89.80% | 0.002 | Random | 0.866（0.609-1.232） |
| AA+ACvsCC |  | | 83.90% | 0.013 | Random | 0.954（0.742-1.226） |
| AA+CCvsAC |  | | 0.00% | 0.954 | Fixed | 0.976（0.765-1.246） |
| CTLA-4 rs231775 | 970/1577 | |  |  |  |  |
| AvsG* |  | | 74.20% | 0.049 | Random | 1.378（1.221-1.555） |
| AAvsGG* |  | | 46.10% | 0.173 | Fixed | 1.976（1.496-2.611） |
| AGvsGG* |  | | 64.70% | 0.092 | Random | 1.391（1.167-1.658） |
| AAvsAG+GG* |  | | 0.00% | 0.347 | Fixed | 1.668（1.286-2.164） |
| AA+AGvsGG* |  | | 71.10% | 0.063 | Random | 1.491（1.261-1.763） |
| AA+GGvsAG* |  | | 56.30% | 0.130 | Random | 0.831（0.706-0.979） |
| COX-2 -765 | 676/1352 | |  |  |  |  |
| CvsG* |  | | 0.00% | 0.944 | Fixed | 2.439（1.687-3.524） |
| CCvsGG |  | | - | - | - | - |
| CGvsGG* |  | | 0.00% | 0.949 | Fixed | 2.514（1.728-3.657） |
| CCvsCG+GG |  | | - | - | - | - |
| CC+CGvsGG* |  | | 0.00% | 0.949 | Fixed | 2.514（1.728-3.657） |
| CC+GGvsCG* |  | | 0.00% | 0.949 | Fixed | 0.398（0.273-0.579） |
| COX-2 -1290 | 676/1352 | |  |  |  |  |
| GvsA |  | | 0.00% | 0.603 | Fixed | 1.365（0.987-1.887） |
| GGvsAA |  | | - | - | - | - |
| GAvsAA |  | | 0.00% | 0.598 | Fixed | 1.384（0.993-1.927） |
| GGvsGA+AA |  | | - | - | - | - |
| GG+GAvsAA |  | | 0.00% | 0.598 | Fixed | 1.384（0.993-1.927） |
| GG+AAvsGA |  | | 0.00% | 0.598 | Fixed | 0.723（0.519-1.007） |
| COX-2 -1195 | 676/1352 | |  |  |  |  |
| AvsG* |  | | 0.00% | 0.913 | Fixed | 1.288（1.130-1.469） |
| AAvsGG* |  | | 0.00% | 0.900 | Fixed | 1.667（1.280-2.171） |
| AGvsGG |  | | 0.00% | 0.671 | Fixed | 1.259（0.997-1.590） |
| AAvsAG+GG |  | | 0.00% | 0.867 | Fixed | 1.426（1.156-1.759） |
| AA+AGvsGG* |  | | 0.00% | 0.725 | Fixed | 1.384（1.110-1.724） |
| AA+GGvsAG |  | | 0.00% | 0.674 | Fixed | 1.033（0.859-1.243） |
| CDKN2A/B rs3731257 | 5714/12222 | |  |  |  |  |
| TvsC |  | | 0.00% | 0.509 | Fixed | 1.047（0.974-1.124） |
| TTvsCC |  | | 0.00% | 0.649 | Fixed | 1.139（0.959-1.354） |
| TCvsCC |  | | 0.00% | 0.819 | Fixed | 1.018（0.924-1.122） |
| TTvsTC+CC |  | | 0.00% | 0.702 | Fixed | 1.132（0.963-1.329） |
| TT+TCvsCC |  | | 0.00% | 0.651 | Fixed | 1.037（0.945-1.137） |
| TT+CCvsTC |  | | 0.00% | 0.661 | Fixed | 1.004（0.915--1.102） |
| CDKN2A/B rs3731249 | 5714/12222 | |  |  |  |  |
| TvsC* |  | | - | - | Fixed | 0.788（0.654-0.949） |
| TTvsCC |  | | - | - | Fixed | 0.921（0.308-2.753） |
| TCvsCC* |  | | - | - | Fixed | 0.773（0.637-0.940） |
| TTvsTC+CC |  | | - | - | Fixed | 0.938（0.314-2.801） |
| TT+TCvsCC* |  | | - | - | Fixed | 0.777（0.641-0.942） |
| TT+CCvsTC* |  | | - | - | Fixed | 1.293（1.064-1.571） |
| CDKN2A/B rs3731239 | 5714/12222 | |  |  |  |  |
| CvsT |  | | 36.10% | 0.211 | Fixed | 1.000（0.934-1.071） |
| CCvsTT |  | | 0.00% | 0.616 | Fixed | 1.029（0.884-1.198） |
| CTvsTT |  | | 0.00% | 0.939 | Fixed | 1.085（0.934-1.261） |
| CCvsCT+TT |  | | 46.60% | 0.171 | Fixed | 0.976（0.889-1.071） |
| CC+CTvsTT |  | | 0.00% | 0.699 | Fixed | 1.059（0.918-1.221） |
| CC+TTvsTC |  | | 45.00% | 0.177 | Fixed | 0.953（0.868-1.045） |
| CDKN2A/B rs3731211 | 5714/12222 | |  |  |  |  |
| AvsT* |  | | 0.00% | 0.555 | Fixed | 1.092（1.012-1.177） |
| AAvsTT* |  | | 78.00% | 0.033 | Random | 1.201（1.001-1.441） |
| ATvsTT |  | | 83.60% | 0.014 | Random | 1.121（0.931-1.351） |
| AAvsAT+TT |  | | 0.00% | 0.826 | Fixed | 1.098（0.999-1.208） |
| AA+ATvsTT |  | | 80.60% | 0.022 | Random | 1.169（0.980-1.396） |
| AA+TTvsAT |  | | 37.60% | 0.205 | Fixed | 1.052（0.954-1.159） |
| CDKN2A/B rs3218009 | 5714/12222 | |  |  |  |  |
| GvsC* |  | | - | - | Fixed | 0.888（0.793-0.995） |
| GGvsCC |  | | - | - | Fixed | 0.648（0.402-1.045） |
| GCvsCC |  | | - | - | Fixed | 0.916（0.807-1.038） |
| GGvsGC+CC |  | | - | - | Fixed | 0.658（0.409-1.061） |
| GG+GCvsCC |  | | - | - | Fixed | 0.897（0.793-1.014） |
| GG+CCvsGC |  | | - | - | Fixed | 1.086（0.958-1.231） |
| CDKN2A/B rs3217992 | 5714/12222 | |  |  |  |  |
| AvsG* |  | | 0.0%% | 0.759 | Fixed | 1.129（1.058-1.205） |
| AAvsGG* |  | | 0.00% | 0.464 | Fixed | 1.261(1.104-1.440) |
| AGvsGG* |  | | 71.50% | 0.061 | Random | 1.130(1.019-1.254) |
| AAvsAG+GG* |  | | 0.00% | 0.557 | Fixed | 1.186(1.056-1.331) |
| AA+AGvsGG* |  | | 47.60% | 0.167 | Fixed | 1.168(1.059-1.288) |
| AA+GGvsAG |  | | 78.00% | 0.033 | Random | 0.968(0.884-1.061) |
| CDKN2A/B rs3217986 | 5714/12222 | |  |  |  |  |
| CvsA |  | | 0.00% | 0.941 | Fixed | 1.008（0.895-1.134） |
| CCvsAA |  | | - | - | Fixed | 0.819(0.447-1.499) |
| CAvsAA |  | | - | - | Fixed | 0.797(0.431-1.474) |
| CCvsCA+AA |  | | 0.00% | 0.916 | Fixed | 1.017（0.897-1.154） |
| CC+CAvsAA |  | | - | - | Fixed | 0.816(0.446-1.493) |
| CC+AAvsCA |  | | 0.00% | 0.891 | Random | 1.027(0.904-1.167) |
| CDKN2A/B rs2811710 | 5714/12222 | |  |  |  |  |
| TvsC |  | | 0.00% | 0.840 | Fixed | 1.068（0.994-1.147） |
| TTvsCC |  | | 0.00% | 0.471 | Fixed | 1.151(0.985-1.346) |
| TCvsCC |  | | 0.00% | 0.356 | Fixed | 1.102(0.944-1.285) |
| TTvsTC+CC |  | | 0.00% | 0.878 | Fixed | 1.071(0.971-1.183) |
| TT+TCvsCC |  | | 0.00% | 0.415 | Fixed | 1.126(0.973-1.303) |
| TT+CCvsTC |  | | 0.00% | 0.477 | Fixed | 1.014(0.920-1.118) |
| CDKN2A/B rs2811708 | 5714/12222 | |  |  |  |  |
| TvsG* |  | | 0.00% | 0.361 | Fixed | 1.118（1.035-1.208） |
| TTvsGG |  | | 0.00% | 0.416 | Fixed | 1.217(0.996-1.485) |
| TGvsGG |  | | 57.60% | 0.125 | Random | 1.086(0.885-1.333) |
| TTvsTG+GG* |  | | 45.50% | 0.176 | Fixed | 1.143(1.039-1.257) |
| TT+TGvsGG |  | | 0.00% | 0.330 | Fixed | 1.165(0.958-1.417) |
| TT+GGvsTG* |  | | 70.80% | 0.064 | Random | 1.107(1.004-1.220) |
| CDKN2A/B rs2518719 | 5714/12222 | |  |  |  |  |
| GvsA* |  | | - | - | Fixed | 1.120（1.012-1.239） |
| GGvsAA |  | | - | - | Fixed | 1.183(0.831-1.685) |
| GAvsAA |  | | - | - | Fixed | 1.048(0.729-1.508) |
| GGvsGA+AA* |  | | - | - | Fixed | 1.133(1.013-1.267) |
| GG+GAvsAA |  | | - | - | Fixed | 1.152(0.810-1.639) |
| GG+AAvsGA* |  | | - | - | Fixed | 1.124(1.002-1.262) |
| CDKN2A/B rs11515 | 5714/12222 | |  |  |  |  |
| GvsC |  | | 57.70% | 0.124 | Random | 0.995(0.907-1.092) |
| GGvsCC |  | | - | - | Fixed | 0.806(0.577-1.128) |
| GCvsCC |  | | 53.30% | 0.143 | Random | 1.035(0.930-1.152) |
| GGvsGC+CC |  | | - | - | Fixed | 0.801(0.573-1.118) |
| GG+GCvsCC |  | | 55.80% | 0.133 | Random | 1.017(0.916-1.128) |
| GG+CCvsGC |  | | 52.50% | 0.147 | Random | 0.960(0.863-1.068) |
| CDKN2A/B rs1063192 | 5714/12222 | |  |  |  |  |
| AvsG* |  | | 21.10% | 0.260 | Fixed | 0.904(0.844-0.968) |
| AAvsGG* |  | | 1.00% | 0.315 | Fixed | 0.832(0.718-0.964) |
| AGvsGG* |  | | 84.20% | 0.012 | Random | 0.895(0.809-0.991) |
| AAvsAG+GG* |  | | 45.20% | 0.177 | Fixed | 0.870(0.759-0.998) |
| AA+AGvsGG* |  | | 71.40% | 0.061 | Random | 0.879(0.799-0.968) |
| AA+GGvsAG |  | | 87.40% | 0.005 | Random | 1.058（0.963-1.162） |
| ABO rs657152 | 920/1735 | |  |  |  |  |
| GvsT* |  | | 0.00% | 0.339 | Fixed | 0.843（0.754-0.944） |
| GGvsTT* |  | | 0.00% | 0.364 | Fixed | 0.742（0.591-0.932） |
| GTvsTT |  | | 0.00% | 0.542 | Fixed | 1.010（0.819-1.247） |
| GGvsGT+TT* |  | | 0.00% | 0.502 | Fixed | 0.737（0.621-0.875） |
| GG+GTvsTT |  | | 0.00% | 0.403 | Fixed | 0.893（0.732-1.089） |
| GG+TTvsGT* |  | | 0.00% | 0.936 | Fixed | 0.824（0.703-0.965） |
| ABO rs505922 | 2317/7269 | |  |  |  |  |
| TvsC* |  | | 60.10% | 0.057 | Random | 0.892（0.830-0.958） |
| TTvsCC* |  | | 58.70% | 0.064 | Random | 0.832（0.716-0.967） |
| TCvsCC |  | | 0.00% | 0.500 | Fixed | 1.037（0.898-1.196） |
| TTvsTC+CC* |  | | 50.90% | 0.106 | Random | 0.814（0.733-0.904） |
| TT+TCvsCC |  | | 33.60% | 0.211 | Fixed | 0.944（0.824-1.081） |
| TT+CCvsTC* |  | | 0.00% | 0.858 | Fixed | 0.854（0.773-0.944） |
| ABO rs495828 | 2317/7269 | |  |  |  |  |
| GvsT* |  | | 48.10% | 0.165 | Fixed | 0.792（0.697-0.900） |
| GGvsTT* |  | | 34.70% | 0.216 | Fixed | 0.721（0.523-0.994） |
| GTvsTT |  | | 0.00% | 0.425 | Fixed | 0.979（0.705-1.359） |
| GGvsGT+TT* |  | | 14.90% | 0.278 | Fixed | 0.735（0.626-0.862） |
| GG+GTvsTT |  | | 21.40% | 0.259 | Fixed | 0.815（0.596-1.115） |
| GG+TTvsGT* |  | | 0.00% | 0.584 | Fixed | 0.764（0.649-0.900） |
| TNF-α rs1800629 | 536/2986 | |  |  |  |  |
| AvsG |  | | 0.00% | 0.888 | Fixed | 1.097（0.892-1.350） |
| AAvsGG |  | | 0.00% | 0.93 | Fixed | 1.467（0.741-2.904） |
| AGvsGG |  | | 0.00% | 0.92 | Fixed | 1.046（0.823-1.330） |
| AAvsAG+GG |  | | 0.00% | 0.929 | Fixed | 1.465（0.742-2.889） |
| AA+AGvsGG |  | | 0.00% | 0.911 | Fixed | 1.077（0.854-1.357） |
| AA+GGvsAG |  | | 0.00% | 0.907 | Fixed | 0.966（0.760-1.227） |
| TERT rs401681 | 1460/7383 | |  |  |  |  |
| TvsC* |  | | 0.00% | 0.758 | Fixed | 1.174(1.108-1.245) |
| TTvsCC* |  | | 0.00% | 0.558 | Fixed | 1.383(1.228-1.557) |
| TCvsCC* |  | | 0.00% | 0.861 | Fixed | 1.170(1.066-1.285) |
| TTvsTC+CC* |  | | 14.10% | 0.322 | Fixed | 1.243(1.123-1.377) |
| TT+TCvsCC* |  | | 0.00% | 0.943 | Fixed | 1.227(1.124-1.340) |
| TT+CCvsTC |  | | 0.00% | 0.891 | Fixed | 0.959(0.884-1.041) |
| TERT rs2853677 | 2916/9481 | |  |  |  |  |
| AvsG* |  | | 9.90% | 0.292 | Fixed | 1.153（1.070-1.244） |
| AAvsGG* |  | | 0.00% | 0.414 | Fixed | 1.331（1.138-1.557） |
| AGvsGG |  | | 0.00% | 0.715 | Fixed | 1.138（0.983-1.318） |
| AAvsAG+GG* |  | | 53.10% | 0.144 | Random | 1.212（1.083-1.356） |
| AA+AGvsGG* |  | | 0.00% | 0.896 | Fixed | 1.210（1.053-1.391） |
| AA+GGvsAG |  | | 42.90% | 0.186 | Fixed | 1.056(0.950-1.173) |
| MTHFR rs1801133 | 184/387 | |  |  |  |  |
| TvsC* |  | | 91.20% | 0.001 | Random | 1.674(1.337-2.095) |
| TTvsCC* |  | | 88.40% | 0.003 | Random | 2.979(1.844-4.812) |
| TCvsCC* |  | | 78.50% | 0.031 | Random | 1.669(1.163-2.395) |
| TTvsTC+CC* |  | | 81.20% | 0.021 | Random | 2.096(1.397-3.146) |
| TT+TCvsCC* |  | | 88.70% | 0.003 | Random | 1.905(1.355-2.677) |
| TT+CCvsTC |  | | 0.00% | 0.79 | Fixed | 0.867(0.636-1.183) |
| IGF-1 rs2288378 | 1068/2185 | |  |  |  |  |
| CvsT |  | | 0.00% | 0.936 | Fixed | 1.000(0.870-1.150) |
| CCvsTT |  | | 0.00% | 0.356 | Fixed | 0.978(0.683-1.401) |
| CTvsTT |  | | 44.60% | 0.179 | Fixed | 0.957(0.664-1.378) |
| CCvsCT+TT |  | | 0.00% | 0.522 | Fixed | 1.009(0.850-1.197) |
| CC+CTvsTT |  | | 18.60% | 0.268 | Fixed | 0.966(0.681-1.372) |
| CC+TTvsTC |  | | 32.50% | 0.223 | Fixed | 1.018(0.854-1.213) |
| IGF-1 rs5742714 | 1068/2185 | |  |  |  |  |
| CvsG |  | | 30.90% | 0.229 | Fixed | 1.101(0.923-1.312) |
| CCvsGG |  | | 63.50% | 0.098 | Random | 0.975(0.536-1.774) |
| CGvsGG |  | | 0.00% | 0.858 | Fixed | 1.150(0.937-1.411) |
| CCvsCG+GG |  | | 64.80% | 0.092 | Random | 0.930(0.514-1.682) |
| CC+CGvsGG |  | | 0.00% | 0.527 | Fixed | 1.140(0.934-1.391) |
| CC+GGvsCG |  | | 0.00% | 0.993 | Fixed | 0.864(0.704-1.060) |
| HIF-1α rs11549467 | 322/430 | |  |  |  |  |
| AvsG* |  | | 0.00% | 0.418 | Fixed | 3.075(1.981-4.775) |
| AAvsGG |  | | - | - | Fixed | 18.303(0.930-360.191) |
| AGvsGG* |  | | 81.90% | 0.019 | Random | 2.946(1.853-4.683) |
| AAvsAG+GG |  | | - | - | Fixed | 18.894(0.961-371.557) |
| AA+AGvsGG* |  | | 63.40% | 0.098 | Random | 3.142(1.987-4.970) |
| AA+GGvsAG* |  | | 82.90% | 0.016 | Random | 0.343(0.216-0.545) |
| HIF-1α rs11549465 | 322/430 | |  |  |  |  |
| TvsC* |  | | 0.00% | 0.349 | Fixed | 1.765(1.238-2.518) |
| TTvsCC* |  | | - | - | Fixed | 3.394(1.284-8.968) |
| TCvsCC |  | | 90.30% | 0.001 | Random | 1.377(0.903-2.101) |
| TTvsTC+CC* |  | | - | - | Fixed | 4.125(1.568-10.855) |
| TT+TCvsCC* |  | | 78.10% | 0.032 | Random | 1.604(1.081-2.380) |
| TT+CCvsTC |  | | 91.40% | 0.001 | Random | 0.760(0.501-1.154) |

Note: 1.Random stands for Random effect model, Fixed stands for Fixed effect model. When P>was 0.1 and I^2^<50% in the heterogeneity test, heterogeneity was considered to be small, the fixed effect model was used to combine the results, on the contrary, it is considered that the heterogeneity is large and the results are combined by the random effect model. 2.All the gene models with standard * were meaningful models for direct meta-analysis. 3. “–’’ mean the stata software failed to calculate the results may due to lack enough data.
