# Supplementary material for: A systematic review and network meta-analysis of single nucleotide polymorphisms associated with pancreatic cancer risk
Source: Aging (Albany NY). 2020 Nov 20;12(24):25256–74. doi: 10.18632/aging.104128 (PMC7803556; doi:10.18632/aging.104128)
Supplement: Supplementary Information 1 [file aging-12-104128-s004.docx]

**Supplementary Information 1**

**Pubmed：**

Search ((((((((Pancreatic Neoplasm[Title/Abstract]) AND (((((((Single Nucleotide Polymorphisms[Title/Abstract]) OR Nucleotide Polymorphism, Single[Title/Abstract]) OR Nucleotide Polymorphisms, Single[Title/Abstract]) OR Polymorphisms, Single Nucleotide[Title/Abstract]) OR Single Nucleotide Polymorphisms[Title/Abstract]) OR SNPs[Title/Abstract]) OR Single Nucleotide Polymorphism[Title/Abstract]))) OR ((Neoplasms, Pancreas[Title/Abstract]) AND (((((((Single Nucleotide Polymorphisms[Title/Abstract]) OR Nucleotide Polymorphism, Single[Title/Abstract]) OR Nucleotide Polymorphisms, Single[Title/Abstract]) OR Polymorphisms, Single Nucleotide[Title/Abstract]) OR Single Nucleotide Polymorphisms[Title/Abstract]) OR SNPs[Title/Abstract]) OR Single Nucleotide Polymorphism[Title/Abstract]))) OR ((Pancreas Cancer[Title/Abstract]) AND (((((((Single Nucleotide Polymorphisms[Title/Abstract]) OR Nucleotide Polymorphism, Single[Title/Abstract]) OR Nucleotide Polymorphisms, Single[Title/Abstract]) OR Polymorphisms, Single Nucleotide[Title/Abstract]) OR Single Nucleotide Polymorphisms[Title/Abstract]) OR SNPs[Title/Abstract]) OR Single Nucleotide Polymorphism[Title/Abstract]))) OR ((Cancers, Pancreas[Title/Abstract]) AND (((((((Single Nucleotide Polymorphisms[Title/Abstract]) OR Nucleotide Polymorphism, Single[Title/Abstract]) OR Nucleotide Polymorphisms, Single[Title/Abstract]) OR Polymorphisms, Single Nucleotide[Title/Abstract]) OR Single Nucleotide Polymorphisms[Title/Abstract]) OR SNPs[Title/Abstract]) OR Single Nucleotide Polymorphism[Title/Abstract]))) OR ((Pancreatic Cancer[Title/Abstract]) AND (((((((Single Nucleotide Polymorphisms[Title/Abstract]) OR Nucleotide Polymorphism, Single[Title/Abstract]) OR Nucleotide Polymorphisms, Single[Title/Abstract]) OR Polymorphisms, Single Nucleotide[Title/Abstract]) OR Single Nucleotide Polymorphisms[Title/Abstract]) OR SNPs[Title/Abstract]) OR Single Nucleotide Polymorphism[Title/Abstract]))) OR ((Cancers, Pancreatic[Title/Abstract]) AND (((((((Single Nucleotide Polymorphisms[Title/Abstract]) OR Nucleotide Polymorphism, Single[Title/Abstract]) OR Nucleotide Polymorphisms, Single[Title/Abstract]) OR Polymorphisms, Single Nucleotide[Title/Abstract]) OR Single Nucleotide Polymorphisms[Title/Abstract]) OR SNPs[Title/Abstract]) OR Single Nucleotide Polymorphism[Title/Abstract]))) OR ((Pancreatic Cancers[Title/Abstract]) AND (((((((Single Nucleotide Polymorphisms[Title/Abstract]) OR Nucleotide Polymorphism, Single[Title/Abstract]) OR Nucleotide Polymorphisms, Single[Title/Abstract]) OR Polymorphisms, Single Nucleotide[Title/Abstract]) OR Single Nucleotide Polymorphisms[Title/Abstract]) OR SNPs[Title/Abstract]) OR Single Nucleotide Polymorphism[Title/Abstract]))
