# Supplementary material for: A systematic review and network meta-analysis of single nucleotide polymorphisms associated with pancreatic cancer risk
Source: Aging (Albany NY). 2020 Nov 20;12(24):25256–74. doi: 10.18632/aging.104128 (PMC7803556; doi:10.18632/aging.104128)
Supplement: Supplementary Materials 1 [file aging-12-104128-s005.docx]

**Supplementary Materials 1**

**Table 1. A network of intergene associations(1).**

| C1 | 0.45 | 1.88 | 0.23 | 2.4 | 5.58 | 0.97 | 0.57 | 0.38 | 0.24 | 1.1 | 1.01 | 0.71 | 2.12 | 0.28 | 2.81 |
| --- | --- | --- | --- | --- | --- | --- | --- | --- | --- | --- | --- | --- | --- | --- | --- |
| 2.2 | C2 | 4.07 | 0.52 | 5.29 | 12.28 | 2.13 | 1.28 | 0.83 | 0.54 | 2.43 | 2.26 | 1.58 | 4.68 | 0.62 | 6.3 |
| 0.53 | 0.25 | C3 | 0.13 | 1.31 | 3 | 0.52 | 0.31 | 0.2 | 0.13 | 0.59 | 0.54 | 0.38 | 1.14 | 0.15 | 1.51 |
| 4.3 | 1.94 | 7.97 | C4 | 10.37 | 23.63 | 4.13 | 2.5 | 1.63 | 1.06 | 4.73 | 4.41 | 3.1 | 9.18 | 1.23 | 12.29 |
| 0.42 | 0.19 | 0.76 | 0.1 | C5 | 2.29 | 0.4 | 0.24 | 0.15 | 0.1 | 0.45 | 0.41 | 0.29 | 0.88 | 0.12 | 1.17 |
| 0.18 | 0.08 | 0.33 | 0.04 | 0.44 | C6 | 0.17 | 0.1 | 0.07 | 0.04 | 0.2 | 0.18 | 0.13 | 0.38 | 0.05 | 0.5 |
| 1.04 | 0.47 | 1.92 | 0.24 | 2.49 | 5.76 | D4 | 0.6 | 0.39 | 0.26 | 1.13 | 1.05 | 0.74 | 2.21 | 0.29 | 2.95 |
| 1.75 | 0.78 | 3.22 | 0.4 | 4.15 | 9.54 | 1.66 | K1 | 0.65 | 0.42 | 1.87 | 1.73 | 1.22 | 3.68 | 0.48 | 4.81 |
| 2.67 | 1.2 | 4.98 | 0.61 | 6.46 | 14.76 | 2.56 | 1.54 | K2 | 0.66 | 2.93 | 2.71 | 1.92 | 5.7 | 0.76 | 7.53 |
| 4.11 | 1.85 | 7.65 | 0.94 | 10.01 | 22.86 | 3.92 | 2.38 | 1.52 | K4 | 4.49 | 4.14 | 2.91 | 8.63 | 1.16 | 11.51 |
| 0.91 | 0.41 | 1.71 | 0.21 | 2.24 | 5.07 | 0.89 | 0.53 | 0.34 | 0.22 | K5 | 0.91 | 0.65 | 1.96 | 0.26 | 2.58 |
| 0.99 | 0.44 | 1.84 | 0.23 | 2.43 | 5.52 | 0.95 | 0.58 | 0.37 | 0.24 | 1.09 | L1 | 0.71 | 2.12 | 0.28 | 2.77 |
| 1.4 | 0.63 | 2.64 | 0.32 | 3.46 | 7.76 | 1.35 | 0.82 | 0.52 | 0.34 | 1.54 | 1.42 | L2 | 3 | 0.39 | 3.93 |
| 0.47 | 0.21 | 0.88 | 0.11 | 1.13 | 2.62 | 0.45 | 0.27 | 0.18 | 0.12 | 0.51 | 0.47 | 0.33 | L3 | 0.13 | 1.32 |
| 3.54 | 1.6 | 6.59 | 0.82 | 8.58 | 19.65 | 3.4 | 2.06 | 1.32 | 0.86 | 3.91 | 3.6 | 2.54 | 7.58 | L4 | 9.99 |
| 0.36 | 0.16 | 0.66 | 0.08 | 0.85 | 1.99 | 0.34 | 0.21 | 0.13 | 0.09 | 0.39 | 0.36 | 0.25 | 0.76 | 0.1 | L5 |

Note:

1. The letters in the table represent different genes, and the Numbers after the letters represent different genetic models.For example:C1(XPC rs2607775 in allele gene model)

2. C: XPC rs2607775;D: XPC rs2228001;K: ERCC2 rs13181;L: ERCC1 rs3212986;

3. No.1: allele gene model;No.2: Homozygous genes;No.3: Heterozygous gene model;No.4: Recessive gene model;No.5: Dominant gene model;No.6: Additive gene model.

**Table2. A network of intergene associations(2).**

| V1 | 1.16 (0.52, 2.62) | 0.34 (0.15, 0.76) | 0.86 (0.38, 1.90) | 1.08 (0.54, 2.17) | 1.54 (0.76, 3.09) | 0.38 (0.19, 0.78) | 0.79 (0.39, 1.60) | 2.23 (1.02, 4.93) | 5.86 (2.57, 13.08) | 0.93 (0.41, 2.04) | 0.26 (0.12, 0.57) | 0.57 (0.28, 1.19) | 0.45 (0.22, 0.95) | 1.11 (0.54, 2.30) | 0.18 (0.08, 0.38) | 1.57 (0.76, 3.27) | 0.81 (0.34, 1.92) | 1.14 (0.49, 2.72) | 0.28 (0.12, 0.68) | 2.89 (1.22, 6.85) |
| --- | --- | --- | --- | --- | --- | --- | --- | --- | --- | --- | --- | --- | --- | --- | --- | --- | --- | --- | --- | --- |
| 0.86 (0.38, 1.93) | V2 | 0.30 (0.13, 0.66) | 0.73 (0.32, 1.67) | 0.93 (0.46, 1.89) | 1.33 (0.63, 2.74) | 0.33 (0.16, 0.67) | 0.68 (0.33, 1.39) | 1.92 (0.84, 4.28) | 5.06 (2.18, 11.73) | 0.80 (0.35, 1.79) | 0.23 (0.10, 0.50) | 0.50 (0.24, 1.03) | 0.39 (0.18, 0.82) | 0.96 (0.46, 2.03) | 0.16 (0.07, 0.33) | 1.35 (0.65, 2.83) | 0.70 (0.30, 1.66) | 0.99 (0.40, 2.37) | 0.24 (0.10, 0.59) | 2.49 (1.04, 6.00) |
| 2.92 (1.32, 6.48) | 3.38 (1.52, 7.57) | V4 | 2.50 (1.12, 5.48) | 3.16 (1.56, 6.40) | 4.49 (2.21, 9.16) | 1.12 (0.56, 2.28) | 2.29 (1.13, 4.65) | 6.54 (2.93, 14.47) | 17.10 (7.42, 40.16) | 2.72 (1.21, 6.10) | 0.77 (0.35, 1.68) | 1.67 (0.82, 3.49) | 1.33 (0.62, 2.77) | 3.25 (1.54, 6.85) | 0.52 (0.25, 1.11) | 4.58 (2.17, 9.50) | 2.37 (1.00, 5.58) | 3.33 (1.39, 7.93) | 0.83 (0.35, 2.00) | 8.43 (3.48, 20.26) |
| 1.17 (0.53, 2.61) | 1.36 (0.60, 3.10) | 0.40 (0.18, 0.89) | V6 | 1.27 (0.62, 2.56) | 1.79 (0.88, 3.69) | 0.45 (0.22, 0.91) | 0.92 (0.45, 1.90) | 2.62 (1.17, 5.86) | 6.89 (2.98, 15.54) | 1.09 (0.49, 2.40) | 0.31 (0.14, 0.67) | 0.67 (0.33, 1.42) | 0.53 (0.25, 1.09) | 1.31 (0.62, 2.73) | 0.21 (0.10, 0.44) | 1.84 (0.89, 3.79) | 0.95 (0.40, 2.23) | 1.34 (0.56, 3.18) | 0.33 (0.14, 0.79) | 3.40 (1.39, 8.11) |
| 0.93 (0.46, 1.86) | 1.07 (0.53, 2.18) | 0.32 (0.16, 0.64) | 0.79 (0.39, 1.60) | W1 | 1.42 (0.80, 2.51) | 0.35 (0.20, 0.63) | 0.73 (0.42, 1.26) | 2.06 (1.03, 4.16) | 5.43 (2.57, 11.42) | 0.86 (0.43, 1.74) | 0.24 (0.12, 0.48) | 0.53 (0.30, 0.96) | 0.42 (0.23, 0.76) | 1.03 (0.57, 1.86) | 0.17 (0.09, 0.30) | 1.45 (0.79, 2.60) | 0.75 (0.36, 1.56) | 1.05 (0.50, 2.24) | 0.26 (0.13, 0.55) | 2.66 (1.27, 5.56) |
| 0.65 (0.32, 1.31) | 0.75 (0.36, 1.59) | 0.22 (0.11, 0.45) | 0.56 (0.27, 1.13) | 0.70 (0.40, 1.25) | W2 | 0.25 (0.14, 0.44) | 0.51 (0.29, 0.90) | 1.45 (0.72, 2.98) | 3.80 (1.80, 8.07) | 0.60 (0.30, 1.24) | 0.17 (0.08, 0.34) | 0.37 (0.20, 0.68) | 0.29 (0.16, 0.54) | 0.73 (0.39, 1.32) | 0.12 (0.06, 0.22) | 1.02 (0.55, 1.89) | 0.53 (0.25, 1.10) | 0.74 (0.34, 1.58) | 0.19 (0.09, 0.39) | 1.87 (0.87, 3.97) |
| 2.62 (1.29, 5.27) | 3.03 (1.48, 6.12) | 0.89 (0.44, 1.80) | 2.22 (1.10, 4.53) | 2.83 (1.58, 4.95) | 4.03 (2.27, 7.12) | W4 | 2.05 (1.17, 3.60) | 5.81 (2.90, 11.75) | 15.23 (7.34, 32.26) | 2.42 (1.19, 4.89) | 0.68 (0.34, 1.39) | 1.50 (0.83, 2.71) | 1.18 (0.65, 2.17) | 2.91 (1.60, 5.22) | 0.47 (0.25, 0.86) | 4.09 (2.23, 7.46) | 2.12 (1.02, 4.36) | 2.97 (1.39, 6.43) | 0.74 (0.36, 1.55) | 7.55 (3.59, 16.03) |
| 1.27 (0.62, 2.56) | 1.48 (0.72, 3.06) | 0.44 (0.21, 0.88) | 1.08 (0.53, 2.24) | 1.37 (0.79, 2.37) | 1.96 (1.11, 3.44) | 0.49 (0.28, 0.85) | W6 | 2.84 (1.42, 5.78) | 7.44 (3.54, 15.62) | 1.19 (0.58, 2.38) | 0.33 (0.17, 0.66) | 0.73 (0.40, 1.31) | 0.58 (0.31, 1.06) | 1.42 (0.78, 2.57) | 0.23 (0.13, 0.42) | 1.99 (1.09, 3.64) | 1.03 (0.49, 2.11) | 1.45 (0.68, 3.05) | 0.36 (0.17, 0.76) | 3.67 (1.75, 7.73) |
| 0.45 (0.20, 0.98) | 0.52 (0.23, 1.19) | 0.15 (0.07, 0.34) | 0.38 (0.17, 0.85) | 0.49 (0.24, 0.97) | 0.69 (0.34, 1.39) | 0.17 (0.09, 0.34) | 0.35 (0.17, 0.71) | X1 | 2.62 (1.15, 5.98) | 0.42 (0.19, 0.91) | 0.12 (0.05, 0.25) | 0.26 (0.13, 0.53) | 0.20 (0.10, 0.42) | 0.50 (0.24, 1.02) | 0.08 (0.04, 0.17) | 0.70 (0.34, 1.46) | 0.36 (0.16, 0.83) | 0.51 (0.21, 1.20) | 0.13 (0.05, 0.29) | 1.29 (0.53, 3.09) |
| 0.17 (0.08, 0.39) | 0.20 (0.09, 0.46) | 0.06 (0.02, 0.13) | 0.15 (0.06, 0.34) | 0.18 (0.09, 0.39) | 0.26 (0.12, 0.56) | 0.07 (0.03, 0.14) | 0.13 (0.06, 0.28) | 0.38 (0.17, 0.87) | X2 | 0.16 (0.07, 0.36) | 0.04 (0.02, 0.10) | 0.10 (0.05, 0.21) | 0.08 (0.04, 0.17) | 0.19 (0.09, 0.41) | 0.03 (0.01, 0.07) | 0.27 (0.12, 0.58) | 0.14 (0.06, 0.33) | 0.19 (0.08, 0.48) | 0.05 (0.02, 0.12) | 0.49 (0.20, 1.22) |
| 1.07 (0.49, 2.42) | 1.25 (0.56, 2.82) | 0.37 (0.16, 0.82) | 0.92 (0.42, 2.04) | 1.17 (0.57, 2.35) | 1.66 (0.81, 3.38) | 0.41 (0.20, 0.84) | 0.84 (0.42, 1.73) | 2.40 (1.10, 5.38) | 6.31 (2.77, 14.52) | X4 | 0.28 (0.13, 0.62) | 0.62 (0.29, 1.29) | 0.49 (0.23, 1.03) | 1.20 (0.57, 2.53) | 0.19 (0.09, 0.41) | 1.68 (0.80, 3.54) | 0.87 (0.37, 2.07) | 1.22 (0.51, 2.97) | 0.31 (0.13, 0.75) | 3.11 (1.31, 7.44) |
| 3.79 (1.76, 8.30) | 4.42 (2.00, 9.84) | 1.30 (0.59, 2.85) | 3.25 (1.49, 7.22) | 4.10 (2.10, 8.18) | 5.86 (2.90, 11.90) | 1.46 (0.72, 2.94) | 2.99 (1.50, 6.04) | 8.49 (3.96, 18.59) | 22.25 (9.92, 50.87) | 3.53 (1.62, 7.79) | X6 | 2.19 (1.08, 4.55) | 1.73 (0.83, 3.51) | 4.24 (2.09, 8.77) | 0.68 (0.33, 1.42) | 5.95 (2.89, 12.34) | 3.09 (1.30, 7.19) | 4.35 (1.82, 10.38) | 1.08 (0.47, 2.59) | 11.00 (4.62, 26.24) |
| 1.75 (0.84, 3.56) | 2.02 (0.97, 4.24) | 0.60 (0.29, 1.21) | 1.49 (0.70, 3.06) | 1.88 (1.04, 3.37) | 2.68 (1.47, 4.98) | 0.67 (0.37, 1.21) | 1.36 (0.76, 2.48) | 3.89 (1.90, 7.90) | 10.20 (4.76, 22.03) | 1.61 (0.77, 3.42) | 0.46 (0.22, 0.93) | a1 | 0.79 (0.46, 1.37) | 1.94 (1.13, 3.34) | 0.31 (0.18, 0.54) | 2.72 (1.58, 4.73) | 1.41 (0.71, 2.81) | 1.98 (0.99, 4.12) | 0.49 (0.25, 1.00) | 5.00 (2.47, 10.15) |
| 2.20 (1.05, 4.60) | 2.55 (1.22, 5.42) | 0.75 (0.36, 1.61) | 1.87 (0.92, 3.93) | 2.38 (1.32, 4.26) | 3.39 (1.84, 6.38) | 0.85 (0.46, 1.54) | 1.73 (0.94, 3.21) | 4.90 (2.39, 10.22) | 12.90 (5.94, 27.86) | 2.05 (0.97, 4.29) | 0.58 (0.28, 1.21) | 1.27 (0.73, 2.18) | a2 | 2.45 (1.40, 4.29) | 0.40 (0.23, 0.69) | 3.45 (1.99, 6.01) | 1.79 (0.88, 3.54) | 2.51 (1.23, 5.17) | 0.63 (0.32, 1.27) | 6.35 (3.12, 13.00) |
| 0.90 (0.44, 1.85) | 1.04 (0.49, 2.19) | 0.31 (0.15, 0.65) | 0.76 (0.37, 1.60) | 0.97 (0.54, 1.75) | 1.38 (0.76, 2.56) | 0.34 (0.19, 0.63) | 0.71 (0.39, 1.28) | 2.00 (0.98, 4.23) | 5.27 (2.44, 11.34) | 0.83 (0.40, 1.75) | 0.24 (0.11, 0.48) | 0.52 (0.30, 0.89) | 0.41 (0.23, 0.72) | a3 | 0.16 (0.09, 0.28) | 1.40 (0.80, 2.45) | 0.73 (0.36, 1.43) | 1.02 (0.50, 2.07) | 0.26 (0.13, 0.52) | 2.59 (1.27, 5.30) |
| 5.56 (2.64, 11.77) | 6.42 (3.07, 13.55) | 1.91 (0.90, 4.03) | 4.76 (2.29, 9.96) | 6.01 (3.32, 10.93) | 8.55 (4.65, 15.76) | 2.13 (1.16, 3.93) | 4.37 (2.39, 7.96) | 12.45 (5.94, 25.88) | 32.53 (15.05, 71.57) | 5.18 (2.47, 10.89) | 1.46 (0.71, 3.07) | 3.19 (1.86, 5.53) | 2.52 (1.44, 4.44) | 6.17 (3.57, 10.82) | a4 | 8.71 (4.95, 15.30) | 4.51 (2.23, 8.97) | 6.32 (3.05, 13.10) | 1.59 (0.79, 3.24) | 16.06 (7.82, 32.91) |
| 0.64 (0.31, 1.32) | 0.74 (0.35, 1.54) | 0.22 (0.11, 0.46) | 0.54 (0.26, 1.12) | 0.69 (0.38, 1.26) | 0.98 (0.53, 1.82) | 0.24 (0.13, 0.45) | 0.50 (0.27, 0.92) | 1.43 (0.69, 2.97) | 3.76 (1.73, 8.19) | 0.59 (0.28, 1.24) | 0.17 (0.08, 0.35) | 0.37 (0.21, 0.63) | 0.29 (0.17, 0.50) | 0.71 (0.41, 1.25) | 0.11 (0.07, 0.20) | a5 | 0.52 (0.26, 1.05) | 0.73 (0.36, 1.48) | 0.18 (0.09, 0.37) | 1.85 (0.90, 3.75) |
| 1.23 (0.52, 2.95) | 1.43 (0.60, 3.38) | 0.42 (0.18, 1.00) | 1.05 (0.45, 2.50) | 1.34 (0.64, 2.75) | 1.90 (0.91, 4.01) | 0.47 (0.23, 0.98) | 0.97 (0.47, 2.03) | 2.75 (1.20, 6.40) | 7.22 (2.99, 17.97) | 1.14 (0.48, 2.70) | 0.32 (0.14, 0.77) | 0.71 (0.36, 1.41) | 0.56 (0.28, 1.13) | 1.38 (0.70, 2.75) | 0.22 (0.11, 0.45) | 1.93 (0.96, 3.83) | b1 | 1.41 (0.63, 3.14) | 0.35 (0.16, 0.77) | 3.56 (1.62, 8.06) |
| 0.88 (0.37, 2.05) | 1.01 (0.42, 2.51) | 0.30 (0.13, 0.72) | 0.75 (0.31, 1.77) | 0.95 (0.45, 2.01) | 1.36 (0.63, 2.92) | 0.34 (0.16, 0.72) | 0.69 (0.33, 1.46) | 1.96 (0.83, 4.68) | 5.15 (2.10, 12.58) | 0.82 (0.34, 1.95) | 0.23 (0.10, 0.55) | 0.51 (0.24, 1.01) | 0.40 (0.19, 0.82) | 0.98 (0.48, 1.98) | 0.16 (0.08, 0.33) | 1.38 (0.68, 2.79) | 0.71 (0.32, 1.59) | b2 | 0.25 (0.11, 0.55) | 2.55 (1.12, 5.66) |
| 3.52 (1.47, 8.27) | 4.09 (1.69, 9.75) | 1.21 (0.50, 2.84) | 3.02 (1.26, 7.15) | 3.80 (1.82, 7.97) | 5.40 (2.57, 11.44) | 1.35 (0.65, 2.81) | 2.76 (1.31, 5.78) | 7.79 (3.40, 18.50) | 20.58 (8.43, 50.76) | 3.26 (1.33, 7.81) | 0.92 (0.39, 2.15) | 2.02 (1.00, 4.01) | 1.60 (0.79, 3.15) | 3.91 (1.93, 7.88) | 0.63 (0.31, 1.26) | 5.51 (2.70, 10.84) | 2.84 (1.29, 6.18) | 3.98 (1.82, 8.86) | b4 | 10.10 (4.56, 22.28) |
| 0.35 (0.15, 0.82) | 0.40 (0.17, 0.96) | 0.12 (0.05, 0.29) | 0.29 (0.12, 0.72) | 0.38 (0.18, 0.79) | 0.53 (0.25, 1.15) | 0.13 (0.06, 0.28) | 0.27 (0.13, 0.57) | 0.77 (0.32, 1.88) | 2.04 (0.82, 4.90) | 0.32 (0.13, 0.77) | 0.09 (0.04, 0.22) | 0.20 (0.10, 0.41) | 0.16 (0.08, 0.32) | 0.39 (0.19, 0.79) | 0.06 (0.03, 0.13) | 0.54 (0.27, 1.11) | 0.28 (0.12, 0.62) | 0.39 (0.18, 0.90) | 0.10 (0.04, 0.22) | b5 |

Note:

1. The letters in the table represent different genes, and the Numbers after the letters represent different genetic models.For example:V1(ABO rs657152 in allele gene model)

2. V: ABO rs657152;W: ABO rs505922;X: ABO rs495828;V: ABO rs657152;

3. No.1: allele gene model;No.2: Homozygous genes;No.3: Heterozygous gene model;No.4: Recessive gene model;No.5: Dominant gene model;No.6: Additive gene model.

**Table 3. A network of intergene associations(3).**

| N1 | 2.11 | 2.1 | 195.55 | 23.67 | 14.6 | 8.24 | 76.18 |
| --- | --- | --- | --- | --- | --- | --- | --- |
| 0.47 | N3 | 1 | 92.88 | 11.25 | 6.94 | 3.91 | 36.15 |
| 0.48 | 1 | N5 | 93.05 | 11.26 | 6.95 | 3.92 | 36.22 |
| 0.01 | 0.01 | 0.01 | N6 | 0.12 | 0.07 | 0.04 | 0.39 |
| 0.04 | 0.09 | 0.09 | 8.26 | O1 | 0.62 | 0.35 | 3.22 |
| 0.07 | 0.14 | 0.14 | 13.39 | 1.62 | O2 | 0.56 | 5.21 |
| 0.12 | 0.26 | 0.26 | 23.72 | 2.87 | 1.77 | O4 | 9.24 |
| 0.01 | 0.03 | 0.03 | 2.57 | 0.31 | 0.19 | 0.11 | O5 |

Note:

1. The letters in the table represent different genes, and the Numbers after the letters represent different genetic models.For example:N1(ABO rs657152 in allele gene model)

2. N: COX-2 -765;O: COX-2 -1195;

3. No.1: allele gene model;No.2: Homozygous genes;No.3: Heterozygous gene model;No.4: Recessive gene model;No.5: Dominant gene model;No.6: Additive gene model.

**Table 4. A network of intergene associations(4).**

| H1 | 0.79 | 0.53 | 0.67 | 1.38 | 20.04 | 0.76 | 55.61 |
| --- | --- | --- | --- | --- | --- | --- | --- |
| 1.26 | H2 | 0.67 | 0.83 | 1.73 | 25.14 | 0.95 | 69.35 |
| 1.89 | 1.5 | H3 | 1.25 | 2.6 | 37.36 | 1.43 | 104.97 |
| 1.5 | 1.2 | 0.8 | H4 | 2.06 | 29.69 | 1.14 | 82.47 |
| 0.73 | 0.58 | 0.38 | 0.49 | H5 | 14.53 | 0.55 | 40.21 |
| 0.05 | 0.04 | 0.03 | 0.03 | 0.07 | H6 | 0.04 | 2.8 |
| 1.32 | 1.05 | 0.7 | 0.88 | 1.82 | 26.16 | I3 | 73.39 |
| 0.02 | 0.01 | 0.01 | 0.01 | 0.02 | 0.36 | 0.01 | I6 |

Note:

1. The letters in the table represent different genes, and the Numbers after the letters represent different genetic models.For example:H1(MUM1L1-CXorf57 rs379742 in allele gene model)

2. H: MUM1L1-CXorf57 rs379742;I: MORC4 rs12837024;

3. No.1: allele gene model;No.2: Homozygous genes;No.3: Heterozygous gene model;No.4: Recessive gene model;No.5: Dominant gene model;No.6: Additive gene model.

**Table 5. A network of intergene associations(5).**

| d1 | 1.05 | 81.81 | 110.32 | 1.6 | 0.06 | 0.05 |
| --- | --- | --- | --- | --- | --- | --- |
| 0.96 | d2 | 73.93 | 94.13 | 1.48 | 0.05 | 0.05 |
| 0.01 | 0.01 | d5 | 1.29 | 0.02 | 0 | 0 |
| 0.01 | 0.01 | 0.77 | d6 | 0.01 | 0 | 0 |
| 0.63 | 0.68 | 51.4 | 67.64 | e1 | 0.04 | 0.03 |
| 17.25 | 19.54 | 1510.18 | 1991.75 | 28.43 | e2 | 0.91 |
| 19.38 | 20.97 | 1605.16 | 2071.95 | 30.5 | 1.09 | e4 |

Note:

1. The letters in the table represent different genes, and the Numbers after the letters represent different genetic models.For example:d1(HIF-1α G1790A rs11549467 in allele gene model)

2. d: HIF-1α G1790A rs11549467;e: HIF-1α -C1772T rs11549465;

3. No.1: allele gene model;No.2: Homozygous genes;No.3: Heterozygous gene model;No.4: Recessive gene model;No.5: Dominant gene model;No.6: Additive gene model.

**Table 6. A network of intergene associations(6).**

| P1 | 1.08 (0.00, 493.09) | 1.10 (0.00, 572.64) | 30.85 (0.04, 13336.22) | 625.66 (1.41, 285806.10) | 1695.21 (3.45, 741988.84) | 1.10 (0.00, 488.14) | 196.60 (0.45, 85107.30) | 188.14 (0.47, 85923.74) | 299.07 (0.64, 127798.13) | 77.32 (0.17, 34034.45) | 489.99 (1.23, 227928.49) | 11104.74 (23.74, 12558426.15) | 13083.55 (30.32, 12924434.94) | 12235.68 (29.23, 12425004.99) | 59.57 (0.13, 26968.48) | 30.98 (0.07, 15736.81) | 93.38 (0.22, 44045.32) | 19.34 (0.05, 8735.98) | 125.99 (0.33, 56997.17) | 860.21 (1.94, 391141.80) | 408.55 (0.88, 191357.22) | 509.78 (1.19, 225177.36) |
| --- | --- | --- | --- | --- | --- | --- | --- | --- | --- | --- | --- | --- | --- | --- | --- | --- | --- | --- | --- | --- | --- | --- |
| 0.93 (0.00, 993.67) | P3 | 1.04 (0.00, 882.49) | 28.79 (0.04, 22539.59) | 594.92 (1.31, 563458.75) | 1538.75 (3.85, 1537639.70) | 1.06 (0.00, 911.45) | 178.55 (0.46, 177043.69) | 179.16 (0.42, 161394.89) | 279.89 (0.68, 306439.87) | 68.98 (0.16, 63536.35) | 463.22 (1.14, 437789.40) | 10647.59 (25.80, 21097580.50) | 12157.66 (27.15, 21898212.21) | 11277.02 (28.82, 22296861.47) | 56.03 (0.14, 51799.13) | 29.70 (0.07, 27779.16) | 87.48 (0.20, 81838.97) | 17.96 (0.05, 17663.02) | 119.21 (0.32, 109824.34) | 805.64 (1.99, 719313.81) | 379.84 (0.98, 377557.24) | 470.06 (1.31, 473782.92) |
| 0.91 (0.00, 958.53) | 0.97 (0.00, 807.88) | P5 | 27.55 (0.04, 19889.57) | 579.94 (1.46, 410068.74) | 1498.03 (3.90, 1351023.53) | 1.02 (0.00, 905.32) | 176.54 (0.46, 155391.97) | 173.91 (0.43, 176257.30) | 263.72 (0.67, 282870.19) | 68.44 (0.16, 58374.63) | 452.34 (1.18, 429343.32) | 10030.53 (24.22, 22860395.34) | 11753.97 (22.49, 25741216.94) | 11077.46 (22.39, 22261322.66) | 53.57 (0.13, 45085.16) | 28.91 (0.07, 28305.54) | 83.13 (0.20, 82258.73) | 16.95 (0.05, 17494.75) | 113.24 (0.27, 106240.76) | 766.81 (1.83, 805450.88) | 363.81 (0.90, 364529.33) | 463.01 (1.13, 359256.37) |
| 0.03 (0.00, 25.75) | 0.03 (0.00, 24.22) | 0.04 (0.00, 27.02) | P6 | 20.06 (0.06, 13516.10) | 53.74 (0.15, 38518.62) | 0.04 (0.00, 25.86) | 6.13 (0.02, 4762.71) | 6.03 (0.02, 4643.54) | 9.38 (0.03, 7013.04) | 2.42 (0.01, 1882.96) | 15.97 (0.05, 12285.81) | 366.82 (0.96, 616623.50) | 410.28 (1.11, 757823.18) | 394.61 (1.05, 670788.02) | 1.94 (0.01, 1518.42) | 1.01 (0.00, 726.84) | 3.01 (0.01, 2024.03) | 0.63 (0.00, 421.03) | 4.22 (0.01, 2998.58) | 26.84 (0.08, 22598.46) | 13.14 (0.04, 9709.06) | 16.50 (0.05, 11740.71) |
| 0.00 (0.00, 0.71) | 0.00 (0.00, 0.76) | 0.00 (0.00, 0.68) | 0.05 (0.00, 16.05) | Q1 | 2.70 (0.01, 1068.74) | 0.00 (0.00, 0.68) | 0.30 (0.00, 119.19) | 0.30 (0.00, 111.19) | 0.48 (0.00, 189.02) | 0.12 (0.00, 47.37) | 0.80 (0.00, 305.44) | 17.89 (0.05, 14702.69) | 20.35 (0.05, 19705.51) | 19.33 (0.05, 16135.84) | 0.09 (0.00, 34.95) | 0.05 (0.00, 17.85) | 0.15 (0.00, 57.97) | 0.03 (0.00, 12.30) | 0.21 (0.00, 76.95) | 1.36 (0.00, 581.62) | 0.65 (0.00, 260.11) | 0.82 (0.00, 299.77) |
| 0.00 (0.00, 0.29) | 0.00 (0.00, 0.26) | 0.00 (0.00, 0.26) | 0.02 (0.00, 6.58) | 0.37 (0.00, 138.43) | Q2 | 0.00 (0.00, 0.26) | 0.11 (0.00, 37.29) | 0.11 (0.00, 42.28) | 0.18 (0.00, 67.41) | 0.04 (0.00, 16.71) | 0.30 (0.00, 111.75) | 6.79 (0.02, 5706.22) | 7.69 (0.02, 6660.72) | 7.30 (0.02, 5681.65) | 0.04 (0.00, 13.28) | 0.02 (0.00, 7.74) | 0.06 (0.00, 19.92) | 0.01 (0.00, 4.63) | 0.08 (0.00, 28.00) | 0.51 (0.00, 199.01) | 0.25 (0.00, 92.61) | 0.31 (0.00, 112.22) |
| 0.91 (0.00, 888.57) | 0.94 (0.00, 780.38) | 0.98 (0.00, 874.04) | 27.61 (0.04, 19668.08) | 539.97 (1.48, 469126.68) | 1483.13 (3.83, 1324189.74) | R1 | 172.24 (0.42, 148707.53) | 168.23 (0.46, 132944.45) | 251.16 (0.69, 226039.57) | 68.40 (0.15, 54168.33) | 441.35 (1.26, 403867.29) | 9807.01 (24.46, 21167649.53) | 11469.18 (26.33, 23640147.77) | 10928.74 (24.83, 21285146.76) | 52.45 (0.13, 43986.90) | 27.68 (0.08, 24618.77) | 83.02 (0.23, 68925.10) | 17.00 (0.05, 15628.63) | 114.42 (0.30, 102817.05) | 759.92 (1.93, 625653.53) | 357.88 (0.95, 335202.49) | 442.61 (1.09, 379432.54) |
| 0.01 (0.00, 2.23) | 0.01 (0.00, 2.18) | 0.01 (0.00, 2.15) | 0.16 (0.00, 61.12) | 3.29 (0.01, 1156.88) | 8.85 (0.03, 3259.19) | 0.01 (0.00, 2.40) | S1 | 0.97 (0.00, 356.97) | 1.54 (0.00, 590.02) | 0.39 (0.00, 146.52) | 2.62 (0.01, 914.57) | 60.44 (0.14, 47377.62) | 68.78 (0.18, 54084.03) | 62.27 (0.17, 52652.84) | 0.31 (0.00, 118.70) | 0.16 (0.00, 69.50) | 0.50 (0.00, 178.12) | 0.10 (0.00, 40.62) | 0.67 (0.00, 245.14) | 4.41 (0.01, 1981.19) | 2.09 (0.01, 780.38) | 2.70 (0.01, 1002.44) |
| 0.01 (0.00, 2.13) | 0.01 (0.00, 2.37) | 0.01 (0.00, 2.32) | 0.17 (0.00, 57.31) | 3.30 (0.01, 1142.16) | 8.71 (0.02, 3569.18) | 0.01 (0.00, 2.16) | 1.03 (0.00, 350.41) | S2 | 1.56 (0.00, 589.68) | 0.40 (0.00, 140.84) | 2.65 (0.01, 1036.29) | 57.59 (0.16, 51775.64) | 65.77 (0.19, 65542.22) | 63.54 (0.17, 54138.09) | 0.32 (0.00, 118.36) | 0.16 (0.00, 66.60) | 0.50 (0.00, 199.86) | 0.10 (0.00, 39.43) | 0.67 (0.00, 248.45) | 4.51 (0.01, 1784.49) | 2.15 (0.01, 834.85) | 2.72 (0.01, 1007.27) |
| 0.00 (0.00, 1.57) | 0.00 (0.00, 1.47) | 0.00 (0.00, 1.49) | 0.11 (0.00, 36.79) | 2.07 (0.01, 806.91) | 5.47 (0.01, 2176.59) | 0.00 (0.00, 1.44) | 0.65 (0.00, 240.72) | 0.64 (0.00, 246.35) | S3 | 0.25 (0.00, 89.71) | 1.71 (0.01, 615.76) | 37.81 (0.10, 33720.90) | 43.22 (0.11, 38005.81) | 40.98 (0.10, 38679.06) | 0.20 (0.00, 80.46) | 0.11 (0.00, 44.37) | 0.31 (0.00, 126.42) | 0.07 (0.00, 29.49) | 0.43 (0.00, 168.25) | 2.91 (0.01, 1114.15) | 1.37 (0.00, 500.80) | 1.76 (0.00, 663.31) |
| 0.01 (0.00, 5.87) | 0.01 (0.00, 6.08) | 0.01 (0.00, 6.16) | 0.41 (0.00, 151.40) | 8.39 (0.02, 3093.87) | 22.55 (0.06, 9068.55) | 0.01 (0.00, 6.68) | 2.54 (0.01, 975.12) | 2.53 (0.01, 985.12) | 3.97 (0.01, 1700.37) | S4 | 6.66 (0.02, 2833.64) | 155.08 (0.40, 121613.17) | 177.70 (0.51, 164318.23) | 159.25 (0.43, 147414.40) | 0.81 (0.00, 322.08) | 0.43 (0.00, 166.55) | 1.29 (0.00, 441.79) | 0.26 (0.00, 103.57) | 1.72 (0.00, 649.80) | 11.05 (0.03, 4792.02) | 5.50 (0.01, 2120.67) | 6.88 (0.02, 2643.42) |
| 0.00 (0.00, 0.81) | 0.00 (0.00, 0.88) | 0.00 (0.00, 0.85) | 0.06 (0.00, 19.82) | 1.25 (0.00, 416.92) | 3.32 (0.01, 1294.45) | 0.00 (0.00, 0.79) | 0.38 (0.00, 137.54) | 0.38 (0.00, 141.51) | 0.58 (0.00, 186.03) | 0.15 (0.00, 53.49) | S5 | 22.76 (0.05, 17981.60) | 26.08 (0.07, 21529.01) | 23.95 (0.06, 18813.62) | 0.12 (0.00, 39.63) | 0.06 (0.00, 22.44) | 0.18 (0.00, 68.26) | 0.04 (0.00, 14.20) | 0.25 (0.00, 105.01) | 1.71 (0.00, 612.53) | 0.81 (0.00, 302.69) | 1.03 (0.00, 391.46) |
| 0.00 (0.00, 0.04) | 0.00 (0.00, 0.04) | 0.00 (0.00, 0.04) | 0.00 (0.00, 1.05) | 0.06 (0.00, 20.15) | 0.15 (0.00, 57.52) | 0.00 (0.00, 0.04) | 0.02 (0.00, 6.90) | 0.02 (0.00, 6.31) | 0.03 (0.00, 10.31) | 0.01 (0.00, 2.48) | 0.04 (0.00, 18.79) | T1 | 1.14 (0.00, 752.46) | 1.09 (0.00, 796.16) | 0.01 (0.00, 1.80) | 0.00 (0.00, 1.17) | 0.01 (0.00, 3.06) | 0.00 (0.00, 0.64) | 0.01 (0.00, 4.47) | 0.08 (0.00, 29.62) | 0.04 (0.00, 14.32) | 0.05 (0.00, 17.73) |
| 0.00 (0.00, 0.03) | 0.00 (0.00, 0.04) | 0.00 (0.00, 0.04) | 0.00 (0.00, 0.90) | 0.05 (0.00, 19.51) | 0.13 (0.00, 55.56) | 0.00 (0.00, 0.04) | 0.01 (0.00, 5.52) | 0.02 (0.00, 5.23) | 0.02 (0.00, 9.06) | 0.01 (0.00, 1.96) | 0.04 (0.00, 15.00) | 0.88 (0.00, 750.27) | T4 | 0.94 (0.00, 631.59) | 0.00 (0.00, 1.69) | 0.00 (0.00, 1.04) | 0.01 (0.00, 2.61) | 0.00 (0.00, 0.60) | 0.01 (0.00, 3.37) | 0.07 (0.00, 27.91) | 0.03 (0.00, 12.41) | 0.04 (0.00, 16.72) |
| 0.00 (0.00, 0.03) | 0.00 (0.00, 0.03) | 0.00 (0.00, 0.04) | 0.00 (0.00, 0.95) | 0.05 (0.00, 18.33) | 0.14 (0.00, 59.78) | 0.00 (0.00, 0.04) | 0.02 (0.00, 5.88) | 0.02 (0.00, 5.86) | 0.02 (0.00, 10.13) | 0.01 (0.00, 2.30) | 0.04 (0.00, 17.41) | 0.91 (0.00, 748.28) | 1.06 (0.00, 826.43) | T6 | 0.00 (0.00, 1.74) | 0.00 (0.00, 1.07) | 0.01 (0.00, 2.90) | 0.00 (0.00, 0.60) | 0.01 (0.00, 3.92) | 0.07 (0.00, 26.85) | 0.03 (0.00, 12.03) | 0.04 (0.00, 16.23) |
| 0.02 (0.00, 7.93) | 0.02 (0.00, 7.02) | 0.02 (0.00, 7.46) | 0.51 (0.00, 182.10) | 10.55 (0.03, 4014.80) | 28.09 (0.08, 11368.71) | 0.02 (0.00, 7.62) | 3.20 (0.01, 1179.45) | 3.13 (0.01, 1351.04) | 5.04 (0.01, 1729.07) | 1.24 (0.00, 467.91) | 8.29 (0.03, 3247.85) | 192.26 (0.56, 172051.64) | 215.00 (0.59, 180008.99) | 202.84 (0.58, 155359.76) | U1 | 0.52 (0.00, 211.21) | 1.57 (0.00, 562.16) | 0.32 (0.00, 133.88) | 2.14 (0.01, 781.74) | 14.57 (0.04, 5307.04) | 6.91 (0.02, 2559.74) | 8.71 (0.02, 3300.88) |
| 0.03 (0.00, 14.76) | 0.03 (0.00, 14.58) | 0.03 (0.00, 14.18) | 0.99 (0.00, 319.48) | 19.55 (0.06, 6402.00) | 52.95 (0.13, 19857.75) | 0.04 (0.00, 13.25) | 6.13 (0.01, 2313.19) | 6.08 (0.02, 1990.86) | 9.29 (0.02, 3601.02) | 2.34 (0.01, 906.41) | 15.93 (0.04, 5904.91) | 368.87 (0.86, 315041.00) | 409.58 (0.96, 366332.85) | 390.69 (0.94, 292333.25) | 1.91 (0.00, 643.35) | U2 | 2.96 (0.01, 1120.85) | 0.61 (0.00, 238.18) | 3.95 (0.01, 1513.31) | 27.01 (0.07, 10110.00) | 13.20 (0.03, 4774.38) | 16.38 (0.04, 6037.03) |
| 0.01 (0.00, 4.61) | 0.01 (0.00, 5.06) | 0.01 (0.00, 5.07) | 0.33 (0.00, 109.85) | 6.77 (0.02, 2530.25) | 17.87 (0.05, 6906.20) | 0.01 (0.00, 4.35) | 2.01 (0.01, 771.17) | 2.02 (0.01, 790.33) | 3.20 (0.01, 1229.37) | 0.78 (0.00, 304.44) | 5.45 (0.01, 2053.91) | 117.03 (0.33, 97021.30) | 136.85 (0.38, 119178.30) | 125.49 (0.34, 96262.24) | 0.64 (0.00, 229.13) | 0.34 (0.00, 134.65) | U3 | 0.20 (0.00, 87.28) | 1.33 (0.00, 561.12) | 9.19 (0.02, 3548.26) | 4.30 (0.01, 1610.78) | 5.47 (0.01, 1993.89) |
| 0.05 (0.00, 21.52) | 0.06 (0.00, 19.59) | 0.06 (0.00, 21.32) | 1.60 (0.00, 561.18) | 32.74 (0.08, 11207.34) | 87.25 (0.22, 30031.92) | 0.06 (0.00, 20.68) | 9.89 (0.02, 3980.33) | 9.93 (0.03, 3353.02) | 15.32 (0.03, 5598.03) | 3.85 (0.01, 1440.69) | 26.96 (0.07, 9263.09) | 592.15 (1.56, 479439.66) | 654.11 (1.66, 457529.31) | 619.86 (1.66, 505510.94) | 3.10 (0.01, 1086.89) | 1.65 (0.00, 601.95) | 5.00 (0.01, 1698.44) | U4 | 6.59 (0.02, 2377.87) | 45.25 (0.11, 17519.42) | 21.51 (0.05, 7542.22) | 26.58 (0.06, 10709.08) |
| 0.01 (0.00, 3.07) | 0.01 (0.00, 3.13) | 0.01 (0.00, 3.67) | 0.24 (0.00, 85.65) | 4.84 (0.01, 1737.37) | 13.12 (0.04, 5039.71) | 0.01 (0.00, 3.35) | 1.49 (0.00, 602.54) | 1.49 (0.00, 551.42) | 2.31 (0.01, 943.15) | 0.58 (0.00, 226.96) | 3.93 (0.01, 1499.00) | 87.89 (0.22, 78025.31) | 100.20 (0.30, 83572.57) | 94.70 (0.25, 74146.27) | 0.47 (0.00, 170.50) | 0.25 (0.00, 91.38) | 0.75 (0.00, 242.56) | 0.15 (0.00, 53.23) | U5 | 6.70 (0.02, 2412.49) | 3.13 (0.01, 1245.46) | 4.06 (0.01, 1502.59) |
| 0.00 (0.00, 0.52) | 0.00 (0.00, 0.50) | 0.00 (0.00, 0.55) | 0.04 (0.00, 13.04) | 0.74 (0.00, 249.89) | 1.94 (0.01, 743.01) | 0.00 (0.00, 0.52) | 0.23 (0.00, 82.74) | 0.22 (0.00, 85.41) | 0.34 (0.00, 125.38) | 0.09 (0.00, 30.01) | 0.58 (0.00, 217.50) | 13.17 (0.03, 11135.75) | 15.15 (0.04, 11593.84) | 13.89 (0.04, 11970.07) | 0.07 (0.00, 23.56) | 0.04 (0.00, 13.64) | 0.11 (0.00, 42.70) | 0.02 (0.00, 8.74) | 0.15 (0.00, 57.03) | Y1 | 0.47 (0.00, 185.64) | 0.60 (0.00, 226.40) |
| 0.00 (0.00, 1.14) | 0.00 (0.00, 1.02) | 0.00 (0.00, 1.11) | 0.08 (0.00, 27.06) | 1.53 (0.00, 580.63) | 4.02 (0.01, 1578.76) | 0.00 (0.00, 1.05) | 0.48 (0.00, 187.69) | 0.46 (0.00, 167.84) | 0.73 (0.00, 299.83) | 0.18 (0.00, 67.07) | 1.24 (0.00, 450.82) | 27.58 (0.07, 23304.00) | 31.31 (0.08, 26365.41) | 29.45 (0.08, 25091.26) | 0.14 (0.00, 59.46) | 0.08 (0.00, 32.24) | 0.23 (0.00, 90.11) | 0.05 (0.00, 20.37) | 0.32 (0.00, 124.99) | 2.11 (0.01, 875.67) | Y4 | 1.28 (0.00, 473.11) |
| 0.00 (0.00, 0.84) | 0.00 (0.00, 0.77) | 0.00 (0.00, 0.89) | 0.06 (0.00, 20.71) | 1.22 (0.00, 454.78) | 3.19 (0.01, 1324.83) | 0.00 (0.00, 0.92) | 0.37 (0.00, 141.14) | 0.37 (0.00, 136.35) | 0.57 (0.00, 218.22) | 0.15 (0.00, 54.11) | 0.97 (0.00, 375.48) | 22.06 (0.06, 19495.73) | 24.64 (0.06, 20122.47) | 23.76 (0.06, 22342.21) | 0.11 (0.00, 42.11) | 0.06 (0.00, 24.43) | 0.18 (0.00, 72.11) | 0.04 (0.00, 15.92) | 0.25 (0.00, 103.90) | 1.66 (0.00, 643.65) | 0.78 (0.00, 295.05) | Y6 |

Note:

1. The letters in the table represent different genes, and the Numbers after the letters represent different genetic models.For example:P1(CDKN2A/B rs3731249 in allele gene model)

2. P: CDKN2A/B rs3731249;Q: CDKN2A/B rs3731211;R: CDKN2A/B rs3218009;S: CDKN2A/B rs3217992;T: CDKN2A/B rs2518719;U: CDKN2A/B rs1063192;Y: CDKN2A/B rs1063192

3. No.1: allele gene model;No.2: Homozygous genes;No.3: Heterozygous gene model;No.4: Recessive gene model;No.5: Dominant gene model;No.6: Additive gene model.

**Table 7. Intermodel network analysis of genes (XRCC4 rs2075685).**

| A1 | 0.99 (0.62, 1.59) | 0.36 (0.23, 0.56) | 2.80 (1.82, 4.44) |
| --- | --- | --- | --- |
| 1.01 (0.63, 1.61) | A2 | 0.36 (0.22, 0.58) | 2.87 (1.75, 4.70) |
| 2.80 (1.79, 4.38) | 2.76 (1.71, 4.53) | A4 | 7.91 (5.00, 12.65) |
| 0.36 (0.23, 0.55) | 0.35 (0.21, 0.57) | 0.13 (0.08, 0.20) | A5 |

Note: the Numbers after the letters represent different genetic models. No.1: allele gene model;No.2: Homozygous genes;No.3: Heterozygous gene model;No.4: Recessive gene model;No.5: Dominant gene model;No.6: Additive gene model.

**Table 8. Intermodel network analysis of genes (XRCC1 rs25487).**

| B1 | 2.33 (1.42, 3.99) | 0.42 (0.28, 0.63) | 4.22 (2.61, 7.15) | 0.61 (0.39, 0.92) |
| --- | --- | --- | --- | --- |
| 0.43 (0.25, 0.70) | B2 | 0.18 (0.10, 0.30) | 1.82 (0.99, 3.32) | 0.26 (0.15, 0.43) |
| 2.38 (1.58, 3.63) | 5.54 (3.31, 9.59) | B4 | 10.11 (6.13, 17.39) | 1.45 (0.93, 2.25) |
| 0.24 (0.14, 0.38) | 0.55 (0.30, 1.01) | 0.10 (0.06, 0.16) | B5 | 0.14 (0.08, 0.23) |
| 1.65 (1.09, 2.55) | 3.83 (2.30, 6.68) | 0.69 (0.45, 1.07) | 6.98 (4.26, 12.11) | B6 |

Note: the Numbers after the letters represent different genetic models. No.1: allele gene model;No.2: Homozygous genes;No.3: Heterozygous gene model;No.4: Recessive gene model;No.5: Dominant gene model;No.6: Additive gene model.

**Table 9. Intermodel network analysis of genes (VDR rs2228570).**

| F1 | 0.99 (0.62, 1.60) | 0.34 (0.21, 0.51) | 2.96 (1.93, 4.63) | 1.02 (0.67, 1.55) |
| --- | --- | --- | --- | --- |
| 1.01 (0.63, 1.61) | F2 | 0.34 (0.20, 0.55) | 2.97 (1.80, 5.00) | 1.02 (0.62, 1.68) |
| 2.97 (1.94, 4.68) | 2.95 (1.80, 4.98) | F4 | 8.86 (5.55, 14.57) | 3.03 (1.91, 4.87) |
| 0.34 (0.22, 0.52) | 0.34 (0.20, 0.56) | 0.11 (0.07, 0.18) | F5 | 0.34 (0.21, 0.54) |
| 0.98 (0.64, 1.50) | 0.98 (0.59, 1.62) | 0.33 (0.21, 0.52) | 2.91 (1.85, 4.67) | F6 |

Note: the Numbers after the letters represent different genetic models. No.1: allele gene model;No.2: Homozygous genes;No.3: Heterozygous gene model;No.4: Recessive gene model;No.5: Dominant gene model;No.6: Additive gene model.

**Table 10. Intermodel network analysis of genes (TP53 rs9895829).**

| G1 | 2.01 (1.79, 2.26) | 0.04 (0.03, 0.06) | 2.05 (1.83, 2.30) | 195.76 (174.86, 219.60) |
| --- | --- | --- | --- | --- |
| 0.50 (0.44, 0.56) | G3 | 0.02 (0.01, 0.03) | 1.02 (0.91, 1.15) | 97.44 (86.91, 109.42) |
| 24.61 (17.52, 35.47) | 49.47 (35.45, 71.29) | G4 | 50.59 (36.25, 72.78) | 4821.53 (3447.59, 6940.58) |
| 0.49 (0.43, 0.55) | 0.98 (0.87, 1.10) | 0.02 (0.01, 0.03) | G5 | 95.20 (85.02, 107.14) |
| 0.01 (0.00, 0.01) | 0.01 (0.01, 0.01) | 0.00 (0.00, 0.00) | 0.01 (0.01, 0.01) | G6 |

Note: the Numbers after the letters represent different genetic models. No.1: allele gene model;No.2: Homozygous genes;No.3: Heterozygous gene model;No.4: Recessive gene model;No.5: Dominant gene model;No.6: Additive gene model.

**Table 11. Intermodel network analysis of genes (CTLA-4 rs231775).**

| M1 | 0.60 (0.44, 0.82) | 2.23 (1.66, 2.92) | 0.25 (0.18, 0.33) | 2.83 (2.12, 3.70) | 1.56 (1.19, 2.06) |
| --- | --- | --- | --- | --- | --- |
| 1.68 (1.22, 2.29) | M2 | 3.75 (2.66, 5.17) | 0.42 (0.29, 0.59) | 4.75 (3.40, 6.48) | 2.60 (1.94, 3.64) |
| 0.45 (0.34, 0.60) | 0.27 (0.19, 0.38) | M3 | 0.11 (0.08, 0.15) | 1.27 (0.95, 1.69) | 0.70 (0.53, 0.94) |
| 3.98 (2.99, 5.50) | 2.38 (1.71, 3.40) | 8.91 (6.49, 12.35) | M4 | 11.32 (8.24, 15.65) | 6.25 (4.63, 8.61) |
| 0.35 (0.27, 0.47) | 0.21 (0.15, 0.29) | 0.79 (0.59, 1.05) | 0.09 (0.06, 0.12) | M5 | 0.55 (0.42, 0.75) |
| 0.64 (0.49, 0.84) | 0.38 (0.28, 0.52) | 1.43 (1.07, 1.89) | 0.16 (0.12, 0.22) | 1.82 (1.34, 2.38) | M6 |

Note: the Numbers after the letters represent different genetic models. No.1: allele gene model;No.2: Homozygous genes;No.3: Heterozygous gene model;No.4: Recessive gene model;No.5: Dominant gene model;No.6: Additive gene model.

**Table 12. Intermodel network analysis of genes (VEGF +405 rs2010963).**

| E3 | 1.77 (0.36, 8.49) |
| --- | --- |
| 0.57 (0.12, 2.79) | E6 |

Note: the Numbers after the letters represent different genetic models. No.1: allele gene model;No.2: Homozygous genes;No.3: Heterozygous gene model;No.4: Recessive gene model;No.5: Dominant gene model;No.6: Additive gene model.

**Table 13. Intermodel network analysis of genes (MTHFR rs1801133).**

| c1 | 0.65 (0.09, 3.66) | 2.31 (0.38, 13.93) | 0.22 (0.03, 1.28) | 3.14 (0.51, 18.80) |
| --- | --- | --- | --- | --- |
| 1.54 (0.27, 10.54) | c2 | 3.59 (0.62, 23.95) | 0.34 (0.05, 2.29) | 4.85 (0.82, 33.51) |
| 0.43 (0.07, 2.64) | 0.28 (0.04, 1.60) | c3 | 0.10 (0.01, 0.54) | 1.35 (0.23, 8.31) |
| 4.53 (0.78, 30.83) | 2.91 (0.44, 19.54) | 10.47 (1.86, 67.79) | c4 | 14.13 (2.37, 95.47) |
| 0.32 (0.05, 1.96) | 0.21 (0.03, 1.22) | 0.74 (0.12, 4.34) | 0.07 (0.01, 0.42) | c5 |

Note: the Numbers after the letters represent different genetic models. No.1: allele gene model;No.2: Homozygous genes;No.3: Heterozygous gene model;No.4: Recessive gene model;No.5: Dominant gene model;No.6: Additive gene model.

**Table 14. Intermodel network analysis of genes (FTO rs9939609).**

| J1 | 2.53 (1.29, 4.80) |
| --- | --- |
| 0.40 (0.21, 0.77) | J5 |

Note: the Numbers after the letters represent different genetic models. No.1: allele gene model;No.2: Homozygous genes;No.3: Heterozygous gene model;No.4: Recessive gene model;No.5: Dominant gene model;No.6: Additive gene model.
